# Multilingual Evidence-Based Question-Answering for Stroke Discharge Summaries: Study of Cross-Lingual Heterogeneity in Clinical Reports

**DOI:** 10.2196/96347

**Published:** 2026-08-19

**Authors:** Vojtěch Lanz, Aleksis Datseris, Svetla Boytcheva, Jiří Mayer, Šárka Zikánová, Robert Mikulík, Pavel Pecina

**Affiliations:** 1 Institute of Formal and Applied Linguistics, Faculty of Mathematics and Physics, Charles University, Malostranské náměstí 25, Prague, 118 00, Czech Republic, 420 951 554 278; 2 Graphwise, Sofia, Bulgaria; 3 Computer Informatics, Faculty of Mathematics and Informatics, Sofia University "St. Kliment Ohridski", Sofia, Bulgaria; 4 Department of Linguistic Modelling and Knowledge Processing, Faculty of Mathematics and Informatics, Sofia University "St. Kliment Ohridski", Sofia, Bulgaria; 5 Health Management Institute, Brno, Czech Republic

**Keywords:** clinical question answering, stroke discharge summaries, evidence extraction, multilingual clinical natural language processing, medical large language models

## Abstract

**Background:**

The Registry of Stroke Care Quality (RES-Q) is a health care quality improvement platform used globally. RES-Q collects structured quality-of-care data for patients with stroke, requiring clinicians to manually extract information from electronic health records or documents such as discharge summaries. This process is essential but time-consuming, particularly given the variability, length, and semistructured nature of clinical reports.

**Objective:**

This study aimed to develop and evaluate a multilingual Evidence-Based Question-Answering framework that identifies supporting text spans in clinical reports of patients with stroke and proposes answer suggestions for structured clinical forms, with the goal of reducing clinician workload while preserving full human oversight.

**Methods:**

We conduct a multilingual study using more than 1500 pseudonymized stroke discharge summaries in 5 languages, annotated with question-evidence-answer triplets. Encoder-based language models are used to extract evidence spans from the reports, while generative language models are used to predict normalized form answers based on the extracted evidences. We compare multiple training strategies, such as (1) models trained on reports in a single target language, (2) models trained jointly on reports in different languages, and (3) models trained on original reports combined with cross-lingual data augmentations. We evaluate performance on Evidence Extraction, Answer Prediction, and end-to-end Evidence-Based Question Answering across the 5 languages.

**Results:**

The presented Evidence-Based Question-Answering system achieves 88% end-to-end accuracy in form filling across 5 languages (77% for patient-specific questions and 95% for default or unverifiable items). Evidence Extraction is the primary bottleneck, reaching 85% *F*_1_ and 79% exact match, whereas Answer Prediction based on extracted evidences is more stable, achieving 95% accuracy. The performance varies by question type, and cross-lingual training generally reduces Evidence Extraction performance but has little effect on Answer Prediction. Model performance is influenced more by reporting practices and dataset characteristics than by language itself.

**Conclusions:**

Evidence-Based Question Answering over multilingual stroke discharge summaries enables human-in-the-loop validation and effective answer prediction with moderate computational resources. Evidence Extraction is the main bottleneck, while Answer Prediction is robust across languages and model sizes. The approach supports structured data collection, although generalization to new languages requires target-language training data.

## Introduction

Improving the quality of health care delivery saves lives. Across diverse medical domains, systematic quality monitoring and feedback have been shown to reduce mortality, complications, and unwarranted variation in care by identifying gaps between evidence-based recommendations and real-world practice [1-3]. Despite its proven effectiveness, large-scale adoption of quality improvement programs remains limited. A major barrier is not the lack of clinical guidelines or clinician willingness, but the practical burden of data collection [4].

As clinical documentation increasingly moves into digital formats, physicians typically compose records as unstructured text stored in hospital information systems [5,6]. Current quality registries and audit systems still rely on manual extraction of structured variables from these unstructured documents, which can be lengthy, sometimes several pages long, making the process time-consuming, error-prone, and difficult to scale [7-9]. Many clinicians review these digitized texts during personal time, contributing to overwork and burnout [10,11]. As a result, quality monitoring is often delayed, incomplete, or abandoned, especially in health care systems facing workforce shortages [12,13]. Automating information extraction from clinical documents could reduce health care workers’ workload. This is where natural language processing (NLP) methods can provide valuable assistance [14-16].

However, clinical text is not standard “natural language” in the traditional sense. In addition to domain-specific jargon, Latin terms, and specialized terminology, discharge summaries often contain enumerations, tables, numerical values, abbreviations, and shorthand phrases. Moreover, because these documents are usually written quickly and under time pressure, they frequently contain typographical errors and inconsistencies. These semistructured, lengthy, and often noisy documents therefore pose unique challenges for NLP models [17,18].

Whether relevant information appears in discharge summaries, progress notes, admission reports, or other parts of the electronic health record (EHR) varies widely across hospitals, countries, and clinical cultures. At the same time, clinical documents differ substantially in structure, level of detail, and terminology depending on the specialty, the individual clinician, and the institution [19-21]. Anchoring automated extraction methods to a single document type risks limiting generalizability. Instead, the key requirement is the ability to robustly retrieve clinically relevant information from heterogeneous, unstructured clinical text, regardless of its specific format or origin.

The language used in such texts also varies by hospital, country, and individual physician [19,22,23]. Despite ongoing efforts to construct domain-specific clinical NLP datasets to support clinical NLP research, the sensitive nature of clinical data and patient privacy concerns severely limit the availability of publicly accessible resources, which are predominantly in English when available [24]. As a result, conducting research and developing NLP tools for non-English clinical data remains challenging, since model performance strongly depends on the availability of domain-specific training data [25]. Consequently, various pretrained models have been published, including multilingual models pretrained on general text [26,27], English clinical-domain models [28,29], and multilingual models further pretrained on clinical data [30]. Interestingly, multilingual pretraining can be more beneficial than domain-specific English pretraining even for clinical NLP tasks in English, for which both relevant training data and models already exist [31].

Data protection and privacy are also limitations of manual data extraction, yet they are rarely discussed. Manual registry entry requires a physical person to read and process large volumes of sensitive patient documentation, often outside the original care context. From a legal and ethical perspective, this creates avoidable exposure of personal health data and increases the risk of unauthorized access, secondary use, or data leakage [32,33]. Automation using NLP methods could help mitigate these risks.

However, for the same reason, the use of third-party NLP models, such as ChatGPT (OpenAI) [34], is infeasible, since the data cannot be shared with external parties beyond the hospital’s control. At the same time, hospitals typically lack the computational resources necessary to run or fine-tune large-scale language models [35]. As a result, developing effective tools for hospital environments must account for both model size and input and output length limitations [36], making the direct processing of entire multipage reports inefficient and technically impractical.

Previous research on clinical information extraction from unstructured EHR initially relied on rule-based methods, classical machine learning classifiers, and recurrent neural networks [37-45], with subsequent studies successfully adopting encoder-based models like BERT (Bidirectional Encoder Representations from Transformers) for specific clinical extraction tasks [46,47]. While these approaches can achieve high performance on well-defined, relatively unambiguous variables (such as detecting large-vessel occlusion or silent brain infarcts), their performance often drops when dealing with complex, heterogeneous, or implicitly expressed clinical attributes. To explore alternatives for these more challenging tasks, recent work has tested prompt-based large language model (LLM) inference without task-specific fine-tuning for document-level classification and schema-defined field extraction directly from discharge summaries [48,49]. However, the practical deployment of their findings may still be constrained by privacy requirements, computational resources, and the need to process lengthy clinical documents.

Until NLP models can provide guaranteed accuracy (which they currently cannot), their predictions cannot be relied upon blindly. This limitation is particularly critical in the clinical domain, where we might need to extract life-critical information, and is further complicated by the phenomenon of hallucinations in generative models [50]. Current mitigation strategies include retrieval-augmented generation (RAG), in which the model is provided with relevant context retrieved from documents to improve the quality of generated answers, and human-in-the-loop (HITL) methods, where a human validator supervises the output [51]. However, even with advanced RAG techniques, guaranteed accuracy cannot be achieved, while sole reliance on HITL methods is inefficient; if a human must manually verify every model prediction by searching the entire report for supporting evidence, they could effectively answer the question themselves, undermining the intended benefit of NLP-based assistance.

We propose to combine the strengths of both approaches. Our method generates answers to clinical questions based on clinical documents, while also providing the evidential spans in the text that support each answer. This facilitates more accurate RAG-style generation [52-56] and enables clinicians to quickly validate the model’s output by referring directly to the relevant passages.

Identifying supporting evidence is nontrivial. Within a report, a question may have no text snippets that provide supporting evidence, a single concise span, or multiple complementary spans that need to be combined to determine the correct answer. The number of evidence spans, therefore, varies across questions and reports, making this real-world task more complex than conventional span-based Question-Answering reading comprehension setups [57,58], where encoder-based models have traditionally achieved the strongest performance [31,59,60].

The goal of this work is to design, evaluate, and demonstrate the potential of NLP methods to assist in reviewing clinical texts in different languages, rather than directly replacing clinicians, by providing easily verifiable predictions that improve efficiency without compromising safety or interpretability.

In this study, we limit our analysis to multilingual discharge summaries related to patients with stroke and evaluate model performance on normalized questions whose answers contribute to the Registry of Stroke Care Quality (RES-Q) [61]. RES-Q is a global quality improvement platform that relies on structured variables extracted from routine clinical records to assess and improve stroke care processes worldwide. These data are extracted from discharge summaries written in different languages, and physicians currently review lengthy reports manually to fill structured forms comprising over two hundred normalized questions with predefined answer types (eg, binary, numeric, multiple choice, date-based, and open-ended), creating a substantial workload and limiting scalability.

**Figure 1.**
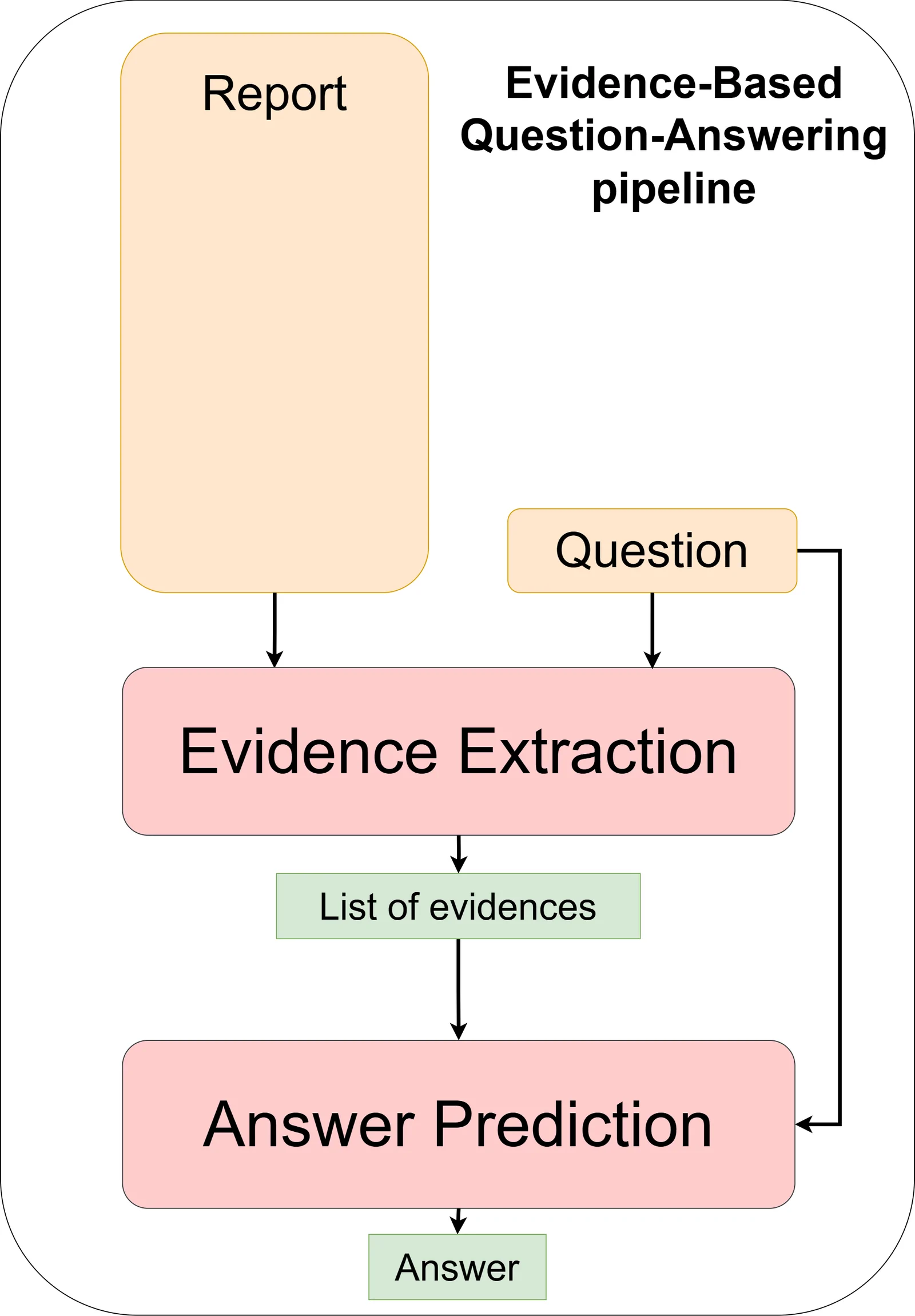
The proposed Evidence-Based Question-Answering pipeline consisting of Evidence Extraction and Answer Prediction components.

The experiments in this study are conducted on the resqEQA dataset (described in the Data section), which comprises 1500 pseudonymized discharge reports of patients with stroke across 5 well-represented languages. The dataset is annotated by clinical experts with RES-Q form responses (answers), along with the corresponding evidence spans in the text. To our knowledge, this is the first work to explore the application of NLP models to this dataset. We also provide an in-depth analysis of how multilinguality and local documentation practices influence model performance in the clinical domain. In particular, we investigate whether a single shared model can be used across all languages or if one model per language is preferable, how data augmentation into other languages impacts performance, whether trained models can be transferred to unseen languages, and, in general, how well this task can be addressed given these real-world constraints. We then decompose this Evidence-Based Question Answering task into two stages, which we explore separately, as visualized in Figure 1:

1. Evidence Extraction: locating text spans within the discharge summary that support the answer to a given question.
2. Answer Prediction: predicting the final answer based on the extracted evidence.

Overall, this work aims to develop and evaluate a multilingual Evidence-Based Question Answering framework that supports structured clinical data extraction from stroke discharge summaries while providing evidence spans in the original text across languages, allowing analysis of cross-lingual differences.

## Methods

### Data

For our experiments, we use the resqEQA dataset, an Evidence-Based Question-Answering dataset in the clinical domain of stroke. The dataset consists of discharge summaries, each paired with a set of question-evidences-answer triplets. Each question is designed for a structured form and therefore lies somewhere between a natural question and a form label. For each question, evidences denote a list of substrings extracted from the given discharge summary context, and the answer represents the final form-compliant response to the question, determined directly from the evidence list (which may contain 0, 1, or multiple elements). The dataset contains 5 question types: boolean (true or false), date-time (date, time, or both), enumeration (multiple-choice with a unique option set), integer, float, and open-ended string. The dataset covers 6 languages: Bulgarian, Greek, English, Spanish, Polish, and Romanian. In total, resqEQA comprises 1596 reports and 181,204 question-evidence-answer triplets, of which 115,203 are impossible cases, that is, questions that have an answer but no supporting text in the discharge summary can be associated with the question, meaning that the answer may be a default value or implicitly inferred from other context. The remaining triplets are possible cases, where at least 1 supporting evidence span is present in the report text. Table 1 presents detailed statistics for each language, including the number of reports, total instances, and the split between possible (containing at least 1 evidence) and impossible (evidence list empty) instances. All languages except Spanish contain hundreds of reports; Spanish therefore serves only as a small reference test set, not sufficient for training.

**Table 1.** Basic statistics of the resqEQA dataset across 6 different languages.

Language	Reports	QEA^a^ triplets	Possible instances	Impossible instances
Bulgarian	286	34,120	11,679	22,441
Greek	303	32,089	15,305	16,784
English	311	28,480	10,313	18,167
Spanish	27	3581	1007	2574
Polish	376	48,952	15,338	33,614
Romanian	293	33,982	12,359	21,623
Total	1596	181,204	66,001	115,203

a QEA: question-evidence-answer.

On average, a single report contains more than 2000 words and nearly 17,000 characters. Additionally, there are structural differences in reports across languages. For example, Bulgarian reports contain fewer lines and paragraphs than other languages, while Spanish reports do not exhibit paragraph structure at all. Polish reports are considerably the longest, whereas English reports are notably the shortest.

**Figure 2.**
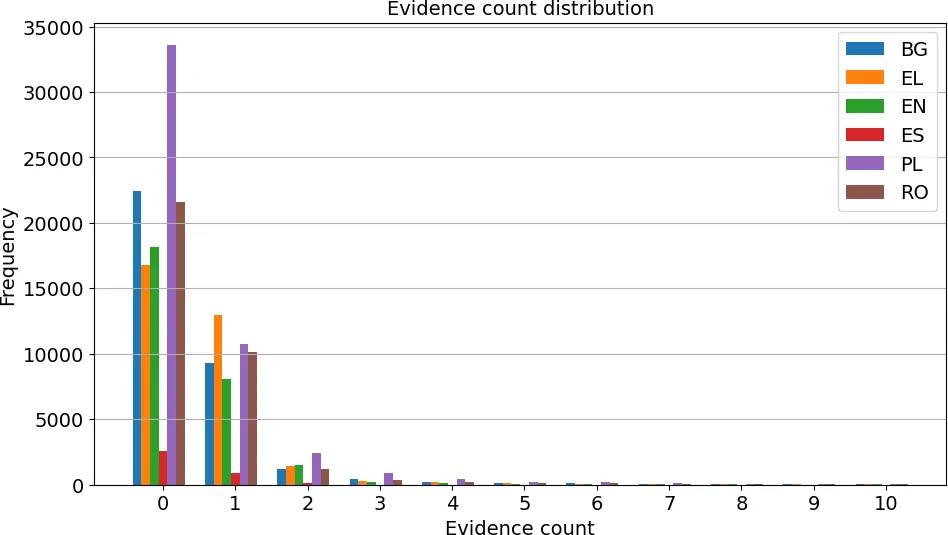
Distribution of the number of evidence per question-evidences-answer instance across all 6 languages in the resqEQA dataset.

A large proportion of question-evidence-answer instances are *impossible*. The full distribution of evidence counts per question per language is illustrated in Figure 2. The distribution follows a Poisson-like pattern: it is uncommon for a question to contain more than 1 or 2 pieces of evidence. However, when evidence for a given question is present, Polish and Spanish evidence segments are, on average, the longest. For Polish, this correlates with the fact that Polish reports are the longest and contain the densest information. Overall, for *possible* questions, an average of approximately 4 words from the report is required to answer a given question. For a detailed breakdown of both report lengths and evidence lengths, refer to Multimedia Appendix 1.

Across the entire *resqEQA* dataset, questions originate from the RES-Q form [61], which contains 238 unique questions, most of which are optional (either irrelevant to the case or not required). Each report therefore includes each form question at most once (either exactly once, or not at all if the question was irrelevant and therefore not annotated). Questions fall into the following categories: anamnesis, onset, admission, diagnosis, treatment, postacute care, discharge, and postdischarge. Each question belongs to one of the predefined types: boolean, integer, number, date-time, enumeration, or open-ended string. The frequency distribution of question types across all reports and languages is presented in Multimedia Appendix 1, where boolean questions dominate, followed by enumeration and integer questions.

**Figure 3.**
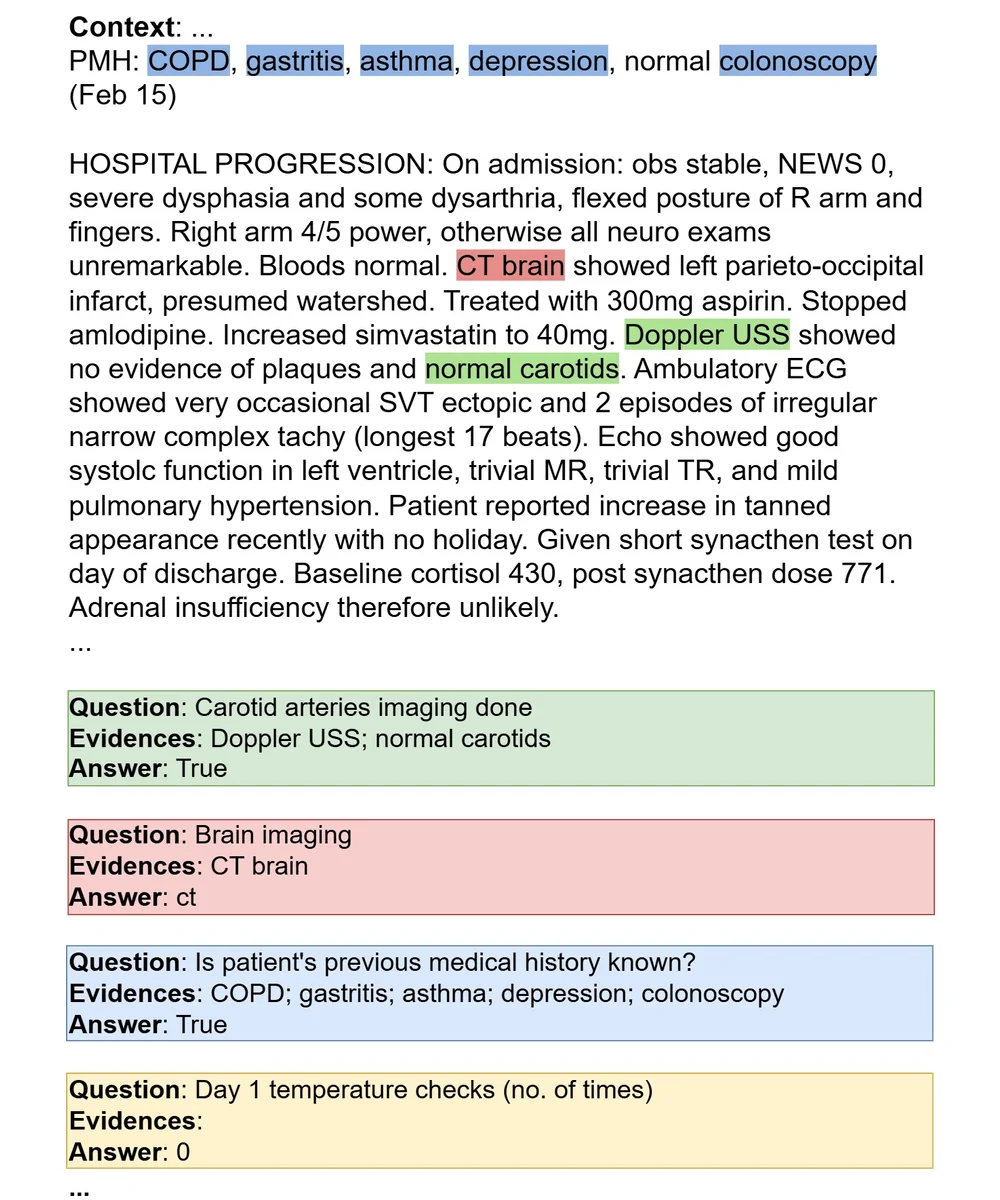
Example of an annotated report paragraph, illustrating sample questions with their answers and supporting evidences. COPD: chronic obstructive pulmonary disorder; CT: computed tomography; ECG: electrocardiography; MR: mitral regurgitation; PMH: past medical history; SVT: supraventricular tachycardia; TR: tricuspid regurgitation; USS: ultrasound scan.

Figure 3 illustrates an example report text, showing a sample of predefined form questions with their answers, where the highlighted texts in the discharge summary indicate evidence mapped to the filled answers in the form.

For our experiments, we used a random split of the train, development, and test subsets for each language such that the test set contains 60 reports per language, the development set contains 30 reports per language, and the remaining reports are placed in the training set. The only exception is Spanish, which is fully included in the test set; therefore, Spanish is not included in the main experiments and is used only as a reference for zero-shot languages.

Evidence and answers for the reports were annotated by clinicians with clinical expertise from different countries on real pseudonymized clinical discharge summary cases. All annotators were provided with identical multipage annotation guidelines that deterministically specified the annotation procedure. All annotators also had access to the same set of questions from a shared questionnaire, which was only translated for specific languages when necessary. Due to the time-consuming nature of the task and the limited availability of physicians, each report was annotated by only 1 clinician. However, interannotator agreement was measured on 10 reports across different languages using 2 independent annotators. On 443 question instances annotated by both annotators, the agreement corresponds to 92.1% accuracy in end-to-end question-answering performance over the final answers. For an additional 139 questions (24% of all annotated questions), annotators disagreed on relevance, meaning these were completed by only 1 of the 2 annotators.

### Task Definitions and Evaluation Metrics

The resqEQA dataset comprises instances of question-evidence-answer triplets. More specifically, each instance contains a report text, a question, a list of supporting evidence, the corresponding form answer, and the list of possible form options. Due to the sensitive and potentially life-critical nature of clinical data, it is important not only to predict the correct form answer but also to identify the precise evidence supporting that answer, enabling clinicians at HITL to easily validate the prediction. In addition, we aim to evaluate how accurately form answers can be predicted based solely on the extracted evidence, as well as to assess the overall performance of the end-to-end prediction pipeline. Accordingly, we define 3 tasks for evaluation, referred to as S1, S2, and S1-S2, where S1 and S2 correspond to the first (Evidence Extraction) and second stage (Answer Prediction) of the full pipeline, respectively, and S1-S2 denotes the end-to-end pipeline combining both stages:

#### S1: Evidence Extraction

Given a pair consisting of a report text (as context) and a question, the task is to identify the minimal list of concise substrings from the report that collectively serve as supporting evidence for answering the question, such that no additional substrings in the report serve as evidence. Correctness is evaluated against the gold evidence list by first concatenating all substrings in the order they appear in the report context, and then comparing the predicted and gold concatenated strings. Evaluation is performed using token-level *F*_1_ and exact match (EM) scores following the SQuAD evaluation script [57]. Tokens from the predicted and gold strings are compared, and *F*_1_ reflects the overlap. If the gold evidence list is empty, a prediction stating that there are no evidence spans is scored as *F*_1_ 100%, otherwise 0%. EM is computed as a strict string comparison: 100% if the predicted and gold strings are identical, and 0% if they differ in any character.

#### S2: Answer Prediction

Given the already correct gold list of evidences, the question, and the set of possible answers, the task is to predict the correct form answer. Accuracy is measured as the proportion of instances where the predicted answer matches the gold answer exactly.

#### S1-S2: Evidence-Based Question Answering

The full Question-Answering pipeline, combining the two previous tasks, is evaluated end-to-end. Given the report context, the question, and the set of possible answers as input, the goal is to predict the correct form answer. Evaluation is performed using accuracy, in the same manner as for the Answer-Prediction task, measured as the proportion of instances where the predicted answer exactly matches the gold answer.

### Proposed Approach

This section presents the methodology proposed in this work for addressing the real-world Evidence-Based Question Answering (S1-S2) task, implemented as a 2-stage pipeline combining Evidence Extraction (S1) and Answer Prediction (S2) for end-to-end answer prediction.

### Evidence Extraction (S1)

Question Answering as an NLP task can take several forms. One variant is comprehensive reading span-based question answering, where the objective is to locate a span within the provided context that answers a given question. Datasets such as SQuAD [57,62] are commonly used for this type of task, containing paragraph contexts with either 0 or 1 answer span. These tasks are typically addressed using encoder-based models [63-66], such as BERT (Google AI) and its variants [26].

Previous works [67] approached span prediction by adding 2 layers on top of the encoder architecture: one for predicting the beginning of the span and another for predicting the end, both trained with cross-entropy loss. If no evidence span exists in the provided context, both the start and end positions are set to the [CLS] token, a special token used in transformer-based language models that marks the beginning of the sequence rather than containing any input content and acts as a summary representation of the entire input sequence [67]. During inference, all valid (start and end) pairs are considered (with start<end, including the [CLS]-[CLS] pair). The score for each candidate span is calculated by multiplying the softmax probabilities of the start and end indices from the 2 layers. The span with the highest score is selected. If the [CLS]-[CLS] pair is chosen, it indicates that no evidence is present in the context for the given question.

Although encoder-based models have become less prevalent compared with generative LLMs, it is crucial to select models based on task suitability rather than prevailing trends. In the present task, the objective is to identify specific evidence spans rather than to generate novel content; the goal is to locate precise textual pointers within existing reports. Accordingly, encoder-based models generally demonstrate higher reliability and performance for this type of task [63-66,68]. Furthermore, generative LLMs require substantially more computational resources and longer inference times, which limits their practical applicability for this task.

Therefore, in our evidence extraction (S1) task, we focus on encoder-based methods. However, long clinical reports pose a challenge, as classical pretrained BERT models are limited to 512 tokens [26], and even long-context models, such as LongFormer (Allen Institute for Artificial Intelligence) [69] or BigBird (Google Research) [70], support only up to 4096 tokens, which is still insufficient in our setting (Multimedia Appendix 1). In addition, reports can contain multiple evidence spans. To address this, we split the report text into chunks of 256 tokens, with a 32-token overlap between neighboring chunks. This overlap prevents evidence from being split across chunk boundaries. Chunking also ensures that most segments contain either 0 or 1 evidence span, which can then be processed using standard methods, as adapted for tasks such as SQuAD, described at the beginning of this section.

**Figure 4.**
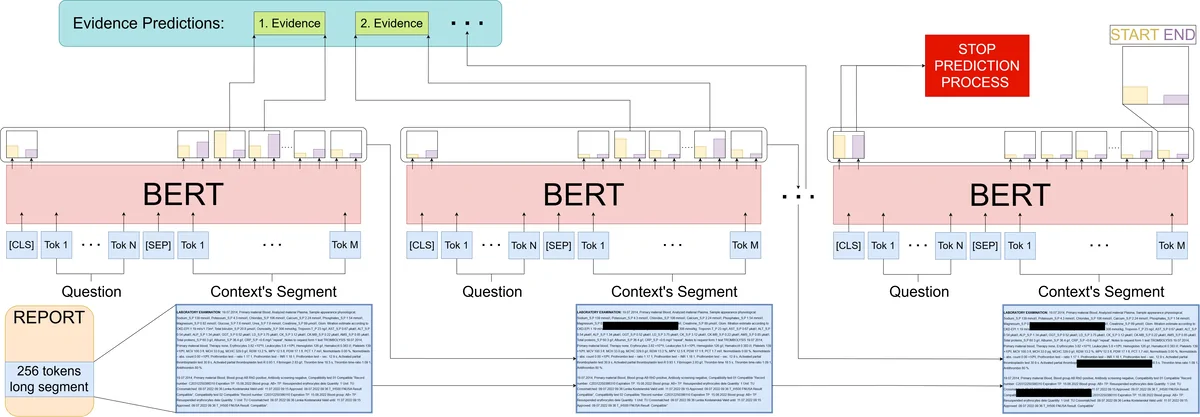
Iterative Evidence Extraction (S1) pipeline for a single context segment and question, repeatedly predicting evidences until the [CLS]–[CLS] span is returned by the model.

But still, some chunks may still contain multiple evidences. To handle these cases, we propose a new iterative method as visualized in Figure 4. The model first identifies the most probable span by maximizing the probability of its start and end positions, comparing it against the probability of the [CLS]-[CLS] span, which indicates no evidence. If the identified span has a probability higher than [CLS]-[CLS], it is stored, masked with a special token, and the process is repeated iteratively until the [CLS]-[CLS] span becomes the most probable prediction. Neighboring or overlapping spans from the same or different chunks are then merged to form complete evidence spans, resulting in 0, 1, or multiple evidence per question and report.

We experiment with and compare general-domain multilingual models, including Multilingual BERT (mBERT) [26], XLM-Roberta (XLMR) [71], and Multilingual ModernBERT (mmBERT) [72], selected for their combination of multilingual coverage, established strong performance on span-based question-answering tasks, and availability as pretrained encoders. To the best of our knowledge, no publicly available encoder-based models are pretrained for both clinical and multilingual data. Furthermore, previous studies have shown that general-domain multilingual models can outperform clinically pretrained English models on several clinical English tasks [31]. Therefore, for English reports in resqEQA, we additionally compare the English ClinicalBERT [28] with its multilingual nonclinical counterpart, mBERT, and similarly compare ClinicalModernBERT [29] with mmBERT.

### Answer Prediction (S2)

For the Answer Prediction (S2) task, we use the generative models Llama3-8B (Meta AI) [27], Mistral-7B-Instruct-v0.1 (Mistral AI) [73], Phi-3.5-mini-instruct 3.8B (Microsoft) [74], and Gemma3-4B (Google DeepMind) [75], and compare each with its clinically oriented counterpart Med42-8B (M42 Health AI Team) [30], BioMistral-8B [76], MediPhi [77], and MedGemma-4B-IT [78], respectively. These models were selected as the currently available generative models with medical pretraining up to 8B parameters, including the base models used for clinical pretraining, offering a balance of state-of-the-art performance and practical computational feasibility. The objective of the Answer Prediction (S2) is to generate the final answer in its exact predefined normalized form, given the question, the corresponding set of possible answers, and the set of evidences previously extracted from the report context. However, when evaluating this task independently, we rely on gold-annotated evidences instead, both to assess the upper bound of model performance for this component and to simulate scenarios in which a human annotator at HITL has already corrected or verified the predicted evidence list.

**Figure 5.**
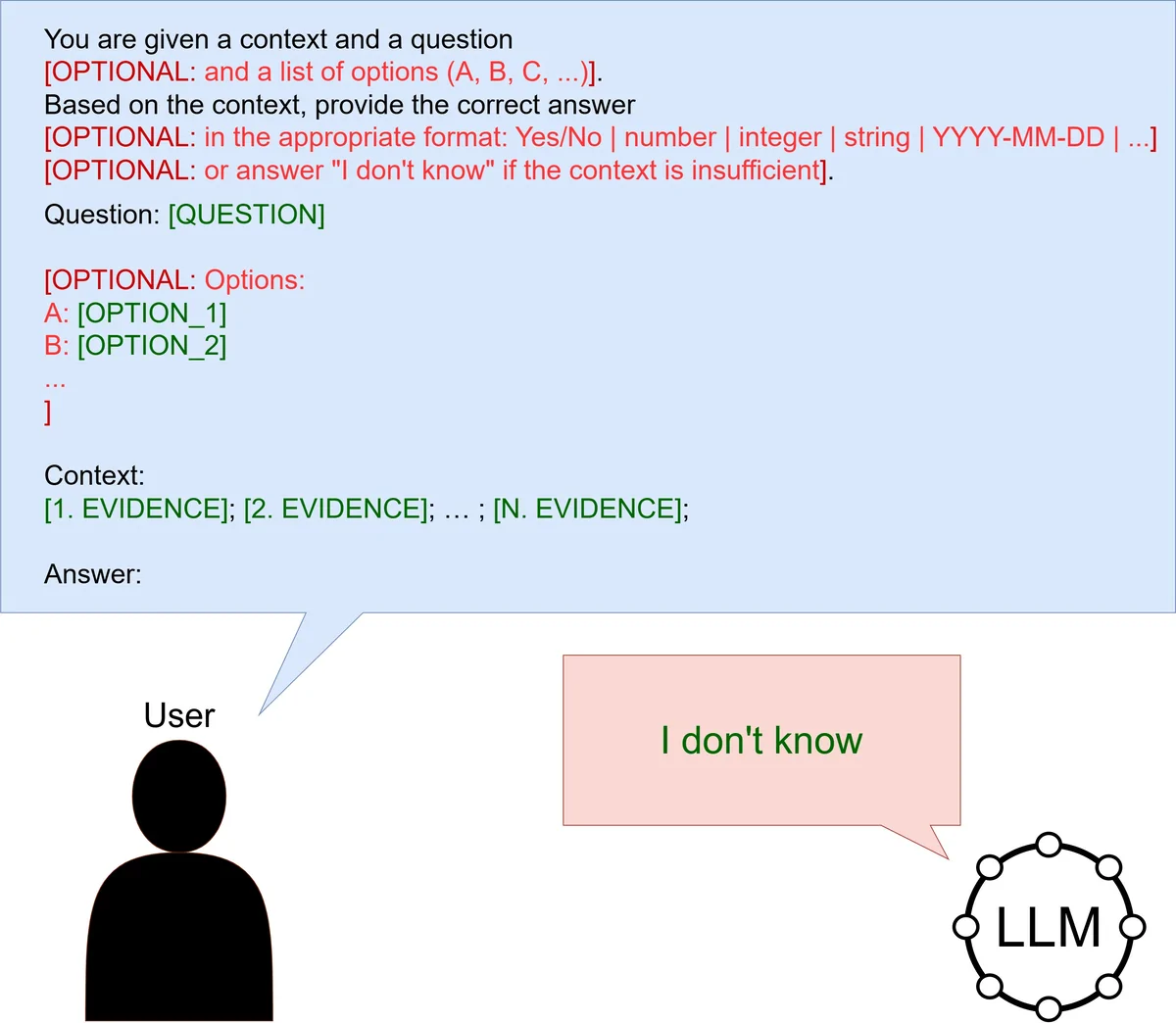
Prompt template for Answer Prediction (S2) given the evidence as context, the question, and the set of possible answers defined by the form specification.

We compare the models first in few-shot (using additional examples of the same question type from the training data in the same language as the target question) and second in fine-tuned settings. The prompt used for Answer Prediction (S2), illustrated in Figure 5, is adapted to the type of the question, whether the required output corresponds to one of the predefined categorical options (A, B, C, …), a boolean value (Yes or No), a numerical value, or another format specified by the form definition. When the question definition allows “None” as a valid option, the prompt also provides the model with the option to answer, “I don’t know.”

Finetuning is performed using LoRA [79] with hyperparameters α=16, *lora_dropout*=0.1, and *r*=64, focusing on the layers “q_proj,” “k_proj,” “v_proj,” “o_proj,” and “out_proj,” with a batch size of 6. Prompts are constrained to a maximum of 512 tokens; in cases where the evidence context is extremely long, it is truncated to fit within this limit. Additionally, 32 tokens are reserved for special tokens and the predicted answer in the final input sequence.

### Evidence-Based Question Answering (S1-S2)

The performance of the full pipeline is evaluated within the RAG framework, as illustrated in Figure 1, by sequentially combining Evidence Extraction (S1) and Answer Prediction (S2). In this setup, Answer Prediction (S2) relies on evidences predicted by the Evidence Extraction (S1) component rather than gold annotations. Specifically, the best-performing Evidence Extraction model is used to generate the list of predicted evidences, which is then fed into the best-performing Answer Prediction model to produce the final answer, assuming no human intervention at any stage. This procedure demonstrates the end-to-end capability of the pipeline. However, for Answer Prediction (S2), although the model is evaluated on predicted evidences to simulate real-world conditions, it is trained using gold evidences.

### Training Configurations

In our experiments, we compare different strategies for combining monolingual and multilingual training. Let the *target language* denote the language being tested, and all remaining languages from the resqEQA dataset are referred to as *other languages*. We then investigate whether a model for the target language performs better when trained solely on that language or additionally on other languages. For the latter, we use either augmented reports automatically translated via machine translation into other languages from the original target-language reports (following the augmentation pipeline of Lanz and Pecina [31]) or the original reports in other languages themselves.

We also aim to investigate how variations in reports affect model performance. These variations may arise not only from differences in language but also from differences in country of origin. While multilingual augmentation has been shown to be beneficial in several tasks [80,81], it remains unclear whether training on reports from different countries, even in different languages, may introduce inconsistencies due to structural differences, writing style, or local conventions.

In addition, we are interested in determining how essential the original reports in the target language are, and whether it is possible to support new languages in a zero-shot setting for which we have no training data.

Therefore, we describe the different sets of training data whose combinations we explore (specifically those combinations described in Multimedia Appendix 2):

- Target original, ***T***: Original reports written in the target language. For instance, when Bulgarian is the target language under test, only the original Bulgarian training reports are used.
- Other original, ***O***: Original reports written in all other languages. For instance, when Bulgarian is the target language under test, we use the original Greek, English, Polish, and Romanian training reports.
- Target-to-other augmented, **$T\overset{aug}{\to }O$**: Reports originally written in the target language, augmented into all other languages. For instance, when Bulgarian is the target language under test, we use augmented training reports translated from the original Bulgarian reports into Greek, English, Spanish, Polish, and Romanian.
- Other-to-target augmented, $O\overset{aug}{\to }T$: Reports originally written in other languages, augmented into the target language. For instance, when Bulgarian is the target language under test, we use augmented training reports translated from original reports in Greek, English, Polish, and Romanian into Bulgarian.
- Other-to-other augmented, $O\overset{aug}{\to }O$: Reports originally written in other languages, augmented into other languages. For instance, when Bulgarian is the target language under test, we use augmented training reports translated from original reports in English, Greek, Polish, and Romanian into Greek, English, Spanish, Polish, and Romanian.

Unless stated otherwise, results for these settings are reported for a single run per configuration due to the large number of experiments across multiple target languages and their computational cost. Model performance is averaged across the available languages to obtain a more stable estimate, reflecting the overall consistency and reliability of the results, while outcomes are also reported separately for each language.

### Ethical Considerations

The study protocol was reviewed and approved by the Ethics Committee of the Faculty of Mathematics and Physics, Charles University (approval REC260616). All data used in this study were handled in accordance with applicable ethical and data protection regulations. The dataset was pseudonymized before analysis, and no personally identifiable information was available to the researchers.

The annotation process was guided by predefined clinical guidelines intended to standardize labeling. Given the intended clinical use case, the proposed Evidence-Based Question-Answering (S1-S2) system is designed to operate in a HITL setting, as fully automated use without clinician validation could propagate extraction errors into structured clinical registry entries. However, to evaluate the theoretical impact of skipping this validation step, it is important to contextualize how registry data are used. Unlike systems designed for real-time, direct clinical decision-making for individual patients, platforms like RES-Q serve as tools for retrospective quality auditing and aggregate statistical monitoring. Because these clinical registries focus on large-scale data aggregation, any potentially unvalidated model errors would primarily impact systemic quality conclusions only if the model introduced systematic, nonrandom biases; randomly distributed errors tend to cancel out in the aggregate metrics. Furthermore, even manual data abstraction by clinicians is inherently prone to human error and rarely achieves perfect accuracy [82]. While fully automated deployment remains unacceptable for safety-critical environments where maximum data integrity is required, the actual clinical safety footprint of isolated extraction errors in this specific setting is minimal.

Despite all languages using the same annotation guidelines and the fact that the questions in the form are direct translations that should not introduce bias, clinician annotators work with report text that follows different writing conventions in each language by different clinical annotators, which may significantly influence model performance.

## Results

### Evidence Extraction (S1)

An important question is whether a single shared model trained jointly on all languages using all original training reports (*T+O*) is sufficient, or whether each language requires a separately trained model using only reports written in that target language (*T*). Another important aspect is the extent to which target-language reports are necessary for training and how well the model performs without them (*O*). Therefore, we compare these training settings in Tables 2 and 3, where we report *F*_1_ and EM scores, respectively.

**Table 2.** Evidence Extraction (S1) *F*_1_ performance across different models under various training configurations.

Model	T^a^	O^b^	Bulgarian	Greek	English	Polish	Romanian	Average	Spanish^c^
XLMR^d^									
	✓	×	85.85	86.62	75.85	81.18	83.75	82.65	—
	✓	✓	83.71	82.39	78.34	74.93	78.30	79.53	—
	×	✓	66.91	51.77	63.41	68.19	63.43	62.74	68.82
mBERT^e^									
	✓	×	88.86	87.61	79.46	80.71	82.52	83.83	—
	✓	✓	85.25	78.20	76.39	74.38	74.48	77.74	—
	×	✓	61.37	54.80	63.65	67.88	62.95	62.13	71.91
mmBERT^f^									
	✓	×	90.53	90.12	80.72	81.66	80.80	84.77	—
	✓	✓	87.45	83.90	79.97	75.24	76.71	80.65	—
	×	✓	66.76	55.16	63.48	66.50	63.38	63.06	66.81

a Target language.

b Other language.

c Not included in the average and is intended only as an additional reference (its test set size differs from the others).

d XLMR: XLM-Roberta.

e mBERT: Multilingual BERT (Bidirectional Encoder Representations from Transformers).

f mmBERT: Multilingual ModernBERT.

**Table 3.** Evidence Extraction (S1) exact match performance across different models under various training configurations.

Model	T^a^	O^b^	Bulgarian	Greek	English	Polish	Romanian	Average	Spanish^c^
XLMR^d^							
	✓	×	80.54	79.95	69.67	74.61	77.17	76.39	—
	✓	✓	78.88	75.28	72.59	69.61	72.18	73.71	—
	×	✓	66.15	48.92	63.12	66.78	62.47	61.49	66.88
mBERT^e^							
	✓	×	83.42	80.75	73.07	74.32	76.21	77.55	—
	✓	✓	80.91	71.39	72.25	70.40	70.82	73.15	—
	×	✓	60.48	52.39	63.28	66.95	62.63	61.15	71.13
mmBERT^f^							
	✓	×	85.05	83.46	74.75	75.29	74.26	78.56	—
	✓	✓	81.46	75.41	73.58	71.03	69.98	74.29	—
	×	✓	66.10	53.49	62.92	64.62	61.32	61.69	63.50

a Target language.

b Other language.

c Not included in the average and serves only as an additional reference (its test set size differs from the others).

d XLMR: XLM-Roberta.

e mBERT: Multilingual BERT (Bidirectional Encoder Representations from Transformers).

f mmBERT: Multilingual ModernBERT.

Detailed results for all training configurations, including those augmented with machine-translated data, are provided in Multimedia Appendix 2. This appendix also reports a detailed breakdown of results for possible and impossible instances across all test question instances. This distinction allows us to evaluate not only how accurately the models extract evidences for questions when the corresponding evidence is present in the report (possible instances), but also how reliably they can predict that a report does not contain an answer to a given question (impossible instances).

We observe that data augmentation of all investigated types ($O\overset{aug}{\to }T$, $O\overset{aug}{\to }O$, $T\overset{aug}{\to }O$) can improve suboptimal training configurations, particularly in scenarios where training data in the target language are scarce or when a single multilingual model is desired. However, augmentation does not benefit the best-performing monolingually fine-tuned models trained in the target language–only setting (*T*); on the contrary, it generally leads to a decrease in performance.

All 3 models achieve near-perfect performance in correctly predicting that no relevant supporting evidence exists in the text for impossible questions. The main challenge lies in questions that do have supporting evidence but where the models fail to locate it. For these instances, the models achieve an average *F*_1_-score of over 60% and an EM of around 45%, which remains highly valuable: in nearly 50% of these possible instances, the models are able to identify all supporting evidence exactly. Interestingly, performance varies notably across languages: for Greek and Bulgarian, both *F*_1_ and EM scores are particularly high, whereas for the remaining 3 languages, performance is comparatively lower.

In Multimedia Appendix 3, we further provide a detailed breakdown of performance across different question data types, including Boolean, multiple-choice enumeration, date-time, open-ended string, and number questions. While Boolean, enumeration, and integer questions achieve consistently high scores across languages, date-time, open-ended string, and number questions exhibit substantial variability, highlighting the impact of diverse writing conventions and styles across countries on model performance.

In Multimedia Appendix 4, we provide a detailed error analysis of the evidence extraction task using the mmBERT model.

### Answer Prediction (S2)

Similar to the previous component of our pipeline, evidence extraction (S1), we investigate whether Answer Prediction (S2) can rely on a single shared model trained jointly on all original training reports across all languages (*T+O*), or whether multilingual training introduces interference and it is preferable to train a separate model for each language using only reports written in the target language (*T*). We further investigate what happens when no training reports are available in the target language and the model has to rely solely on reports from the remaining languages (*O*). In addition, we examine whether model size (4B vs 8B parameters) plays a role and whether clinical pretraining is an important factor. Table 4 reports accuracy for different training configurations and models. We observe that, similarly to S1, the best results are obtained in the monolingual setting (*T*), where a separate model is trained for each language. The highest performance is achieved by Med42, reaching an average accuracy of 95%. Nevertheless, the multilingual shared model trained in the *T+O* setting exhibits only a minimal decrease in performance. The absence of reports in the target language has a substantial impact on the results, although this effect is considerably less severe than for the S1 component. Furthermore, model size does not appear to play a major role, as all evaluated models achieve very similar performance. Likewise, clinical pretraining does not appear to provide a particularly strong advantage.

**Table 4.** Answer Prediction (S2) results for models trained in various training settings.

Model	T^a^	O^b^	Bulgarian	Greek	English	Polish	Romanian	Average	Spanish^c^
MediPhi									
	✓	×	92.79	97.03	93.54	92.02	96.12	94.30	—
	✓	✓	92.90	97.21	93.45	91.47	96.83	94.37	—
	×	✓	85.03	91.91	85.38	82.77	92.39	87.49	85.26
Phi3.5 Mini									
	✓	×	92.81	97.25	93.85	91.77	96.40	94.41	—
	✓	✓	92.88	97.39	92.97	91.58	96.76	94.31	—
	×	✓	84.48	94.23	83.85	81.41	92.75	87.34	85.90
MedGemma									
	✓	×	92.54	97.48	93.56	92.33	96.86	94.55	—
	✓	✓	93.14	97.65	93.52	91.83	96.91	94.61	—
	×	✓	86.56	95.32	87.72	85.42	91.96	89.40	86.57
Gemma3									
	✓	×	92.55	98.01	93.72	91.87	96.61	94.55	—
	✓	✓	92.42	97.65	93.14	91.36	96.80	94.27	—
	×	✓	86.23	95.33	87.14	84.47	91.77	88.99	86.51
LLaMA3									
	✓	×	92.95	97.79	94.49	92.71	97.03	94.99	—
	✓	✓	93.18	97.56	94.31	91.73	96.95	94.75	—
	×	✓	85.36	95.74	87.21	82.57	92.39	88.65	84.14
BioMistral									
	✓	×	93.09	97.68	94.80	92.42	97.13	95.02	—
	✓	✓	93.24	97.82	93.30	92.27	97.16	94.76	—
	×	✓	86.37	92.67	86.55	85.67	93.74	89.00	86.85
Mistral									
	✓	×	93.27	97.76	94.57	92.60	97.16	95.07	—
	✓	✓	93.21	97.74	93.34	92.26	97.00	94.71	—
	×	✓	85.14	93.12	86.57	84.45	93.51	88.56	86.26
Med42									
	✓	×	93.04	97.82	94.88	92.75	96.91	95.08	—
	✓	✓	93.06	97.84	94.04	92.06	97.16	94.83	—
	×	✓	86.45	95.36	87.27	84.73	93.38	89.44	86.40

a Target language.

b Other language.

c Not included in the average and serves only as an additional reference (its test set size differs from the others).

Multimedia Appendix 2 provides a detailed breakdown of results obtained using the $T\overset{aug}{\to }O$, $O\overset{aug}{\to }T$, and $O\overset{aug}{\to }O$ augmentation strategies. It also reports separate results for possible and impossible instances only. Similar to the previous case, augmentation has neither a substantial positive nor a negative effect on performance. However, for impossible questions, Greek and Romanian exhibit highly consistent default predictions, achieving near 100% accuracy, whereas for Bulgarian and Polish the model is more frequently surprised, though still maintains strong accuracy above 90%. But also for possible questions, the models demonstrate strong performance, achieving an average accuracy of approximately 93%.

Multimedia Appendix 3 provides a detailed breakdown of results according to the answer data type. Boolean questions are the easiest, and enumeration questions also achieve consistently high accuracy across languages. Other question types show considerable variability and inconsistent performance, reflecting the differences in how reports are written in different countries and languages.

Multimedia Appendix 5 presents the results of the models without fine-tuning, operating solely in a few-shot setting. When considering the 20-shot setting, the models achieve accuracy of up to 90%, indicating that target-language training improves performance by only 2‐3 percentage points.

We already know that Evidence Extraction (S1) can be a bottleneck in the overall pipeline, as we achieve on average only 78% EM with the gold annotations, and it can impose a substantial burden on clinicians in the HITL setting. Identifying or typing the regions of evidence within paragraph-level retrieval settings may be a simpler task [59]. However, this increases the challenge for the generative model used for Answer Prediction (S2), which must operate over longer contexts. It also poses additional difficulty for medical experts in the HITL setting, who need to quickly locate and verify the relevant evidences within retrieved extended text segments. To assess how generative models could potentially handle longer text segments, Multimedia Appendix 6 shows the effect of extended contexts on the Med42 model. We show that substantially longer contexts, up to 25 times longer than the evidence spans themselves, have only a minimal impact on model performance, demonstrating the robustness of generative models and highlighting promising directions for future research.

### Evidence-Based Question Answering (S1-S2)

In the previous sections, we evaluated Evidence Extraction (S1) and Answer Prediction (S2) independently. From the Evidence Extraction (S1) experiments, mmBERT was found to perform best in the *T* setting. For Answer Prediction (S2), the Med42 model exhibited the most stable performance. Both models are trained using gold data; specifically, the Med42 model for Answer Prediction (S2) is trained on gold evidence. During evaluation, however, Med42 is provided with predicted evidences generated by the mmBERT model from the Evidence Extraction module rather than the gold evidences.

Table 5 illustrates a comparison of Answer Prediction (S2) performance using gold evidence from the previous Answer Prediction (S2) section under Results versus Evidence-Based Question-Answering (S1-S2) performance using predicted evidences. The table also includes a baseline based on the prompted LLM gpt-oss-120B [82] operating in a zero-shot setting, where no evidence spans are provided or requested, and no human validator is involved at any stage of the process, only the report text and set of questions. Despite an average drop of approximately 8%, the model still achieves 88.47% accuracy for correctly filling in form questions, with 77.19% accuracy on *possible* questions and 94.91% accuracy on *impossible* questions. This confirms that *possible* questions represent the most challenging aspect of the full pipeline, as their performance drops by nearly 20% compared with gold evidences.

**Table 5.** Evidence-Based Question-Answering (Evidence Extraction [S1]+Answer Prediction [S2]) performance compared with baselines.

Setting	Bulgarian	Greek	English	Polish	Romanian	Average
Full testset						
gpt-oss-120B zero-shot (S1-S2)	77.25	86.58	69.28	79.73	81.99	78.97
Answer Prediction (S2) - gold evidences	93.04	97.82	94.88	92.75	96.91	95.08
Evidence-Based Question-Answering (S1-S2)	89.09	93.87	86.66	83.78	88.96	88.47
Possible instances						
gpt-oss-120B zero-shot (S1-S2)	76.20	83.62	71.15	71.89	73.76	75.32
Answer Prediction (S2) - gold evidences	94.95	95.45	92.03	91.27	94.78	93.70
Evidence-Based Question-Answering (S1-S2)	85.26	88.36	72.29	64.11	75.92	77.19
Impossible instances						
gpt-oss-120B zero-shot (S1-S2)	77.78	89.28	68.19	83.32	86.92	81.10
Answer Prediction (S2) - gold evidences	92.07	99.97	96.55	93.42	98.18	96.04
Evidence-Based Question-Answering (S1-S2)	91.04	98.87	95.09	92.78	96.76	94.91

## Discussion

### Principal Findings

In this study, we show that clinical Question Answering over stroke discharge summaries can be effectively framed as an Evidence-Based Question Answering (S1-S2) problem across 5 languages (Bulgarian, Greek, English, Polish, and Romanian). This formulation exposes an intermediate step, Evidence Extraction (S1), in which the model identifies specific text spans as evidences within the discharge summary, enabling fast HITL validation for stroke registry workflows and also allowing the use of RAG-style modeling without requiring the very large computational resources needed to fit or process entire reports directly in an LLM. Even without evidence HITL validation, the end-to-end system achieves 88% accuracy in form filling, including 77% for patient-specific questions (*possible*) and 95% for default or unverifiable items (*impossible*).

Evidence Extraction (S1), the component responsible for identifying supporting text spans in the report for a given question, proved to be the bottleneck of the overall Evidence-Based Question-Answering (S1-S2) pipeline. Although detecting cases in which no evidence is present for a given question within a report was nearly perfect (ie, for *impossible* questions), extracting the specific supporting spans was substantially more challenging, reaching only 62% *F*_1_ and 45% EM. But still, as a result, the system was able to surface sufficiently relevant text to assist a human validator in roughly half of the cases. After fine-tuning, we observed on English reports that multilingual general-domain models performed even better than clinically pretrained models, suggesting that it is not clear whether clinical pretraining would provide any additional benefit for this component.

Answer Prediction (S2), where the model predicts the final form answer based on extracted and human-validated evidences, was the more stable component of the pipeline. Accuracy reached 95% overall (94% for questions with available evidences and 96% for cases with no evidence), and performance remained robust even when provided with evidence windows more than 20 times longer than the concise spans. This indicates that Answer Prediction (S2) does not require highly focused evidence to generate reliable outputs, though whether this tolerance to noisy input translates to human performance in HITL settings remains an open question. Clinical pretraining does not appear beneficial for this component either, as base models and clinically pretrained counterparts behave similarly. Moreover, smaller models achieve nearly comparable performance, with only a minor drop despite having roughly half the parameter count.

Performance also varied substantially across question types. Boolean and enumeration questions were handled consistently well, whereas date-time, numeric, and open-ended string questions were more error-prone and showed larger discrepancies across languages. These differences correlate with the distribution of question types in the dataset, making it unclear whether the increased difficulty reflects intrinsic task complexity or simply underrepresentation during training.

### Cross-Language Variation

The results indicate that observed differences between languages are primarily due to variations in reporting practices rather than intrinsic language aspects. Discharge summaries from different countries and hospitals varied in length, structure, and narrative detail, as reflected in our dataset statistics, which led to heterogeneous difficulty across languages. Furthermore, certain languages tended to use more consistent default values for *impossible* questions, whereas others exhibited more variable or implicit phrasing, which affected model performance. Similarly, differences in question data types led to varying difficulty across languages, with some types being easier to answer in certain languages than in others.

Multilingual and augmented training had divergent effects across components. For Evidence Extraction (S1), including reports from other languages (or their machine-translation–based augmentations) during training consistently reduced performance, indicating that cross-language mixing introduces noise rather than useful generalization. In contrast, augmenting target-language reports into other languages did not cause any issues, confirming that the challenge lies in report content and writing style rather than the language itself. For Answer Prediction (S2), performance was largely insensitive to multilingual training and comparable across model sizes, suggesting that a single shared model is sufficient for this component.

Generalization to new, unseen languages cannot be guaranteed under current conditions, as both Evidence Extraction (S1) and Answer Prediction (S2) rely on the presence of original target-language reports during training to achieve meaningful performance; without them, accuracy drops substantially.

To better understand the sources of the observed cross-language performance differences, we conducted an additional analysis of the underlying data and identified the following cross-linguistic differences in reporting practices. Our experimental findings suggest that reporting conventions vary substantially across countries and languages and that these differences affect model performance. While we already compared report length and structural differences across languages, we further analyzed the reports to identify specific discrepancies in reporting practices that may contribute to the observed performance variation.

We found that reports in Bulgarian use mmol/L as the scale for cholesterol, whereas other languages predominantly use mg/dL. Although the official scale for annotations was mmol/L, only 46% of Polish annotators converted values from mg/dL to mmol/L. In the remaining languages, no conversion was performed. Additionally, Bulgarian reports use the Glasgow–Liège Coma Scale, while reports from other countries rely on the Glasgow Coma Scale. In English reports, the patient’s age is not explicitly stated. Instead, only the date of birth is provided, requiring the age to be derived using the admission date. In contrast, reports in the other languages explicitly provide the patient’s age.

Date and time annotation conventions also vary significantly across languages, particularly with respect to the inclusion of seconds and the balance between strictly numeric formats and narrative temporal expressions. Bulgarian and Polish reports are distinct in their dual approach and frequently combine natural language phrases such as “the day before” with numeric dates that generally omit seconds. Bulgarian follows a day-first format with dot separators (DD.MM.YYYY HH:mm), whereas Polish adopts the ISO-8601 year-first format with hyphens (YYYY-MM-DD HH:mm). Spanish is the only language in the dataset to explicitly include seconds in its standard notation, allowing both dot- and slash-separated day-first formats (DD.MM.YYYY HH:mm:ss or DD/MM/YYYY HH:mm:ss). The remaining languages rely on day-first numeric timestamps without seconds but differ in formatting details. Romanian favors a 2-digit year (DD.MM.YY HH:mm), Greek shows inconsistency in separators and zero-padding (DD.M.YYYY HH:mm or DD/MM/YYYY HH:mm), and English reports generally follow the European convention using slashes (DD/MM/YYYY HH:mm).

### Practical Implications for Clinical Use

The findings indicate that Evidence-Based Question Answering (S1-S2) can meaningfully support structured data collection from long discharge summaries about stroke patients in multiple languages, especially when paired with HITL validation. Highlighted evidence spans can help speed up verification and support the validation of automatically generated answers against the report. Preliminary internal measurements suggest that the NLP-assisted annotation workflow reduces overall clinician annotation effort by approximately 25% across the full Evidence-Based Question Answering (S1-S2) pipeline.

Answer Prediction (S2), operating on extracted and human-validated evidence, performs very well across languages. For practical deployment, it is advantageous to directly integrate questions for which sufficient training data exists and for which reliable performance is observed, such as Boolean and enumeration questions.

From a computational perspective, Evidence Extraction (S1) requires a separate model for each language; however, the encoder models we use for Evidence Extraction (S1) are relatively small (up to 307M parameters) and easily deployable. In contrast, for Answer Prediction (S2), which uses substantially larger models, a single shared model is sufficient for all languages, with 4B parameters providing strong performance and making larger 8B models unnecessary.

### Limitations

This study has several limitations. Both stages of our Evidence-Based Question Answering (S1-S2) pipeline rely on transformer models that may exceed available computational capacity in some hospital settings. The evaluation covers 6 languages, with a very limited number of reports available for Spanish and limited training instances for some question types. The Spanish dataset (27 reports) is used only as a small-scale test reference and was not part of a fully powered evaluation; zero-shot transfer to Spanish was therefore only explored rather than systematically evaluated. Findings cannot automatically be extended to languages with substantially different reporting structures or insufficient training data.

### Future Directions

Future work may explore retrieval strategies that operate at paragraph or section level to mitigate the sensitivity of span extraction. Lightweight architectures or distillation approaches could improve deployability in resource-constrained environments. Extending the framework to additional clinical domains and languages would help assess broader generalizability. Human-centered evaluations, such as systematic time-motion studies measuring verification time, cognitive load, error correction effort, and clinician trust, would provide direct and rigorous evidence of practical impact in real-world workflows focused on how the system accelerates completion of stroke registries such as RES-Q, where clinicians directly fill standardized form fields without requiring explicit evidence span or other annotation.

More complex cross-language variation analysis and bias auditing across languages may further be useful in the form of measuring differences in the frequency and distribution of evidence spans and relevant sections at different positions in the text, as well as how reporting conventions vary in detail not only between languages but also between reports within the same language across different hospitals and settings.

### Comparison With Previous Work

Although earlier studies did not directly address Evidence-Based Question Answering (S1-S2) in the same sense as we do, various works have focused on clinical information extraction from discharge summaries. For this purpose, the n2c2 shared task [83] was introduced, targeting named entity recognition, concept extraction, relation classification, and end-to-end information extraction systems across different discharge summaries. Subsequent research explored recurrent neural network-based approaches [84], which were outperformed by encoder-based transformer models, such as BERT [85]. The data from this shared task also served as the basis for creating the emrQA dataset [58], which contains synthetic questions aimed at locating explicit evidence spans in text, similar to our Evidence Extraction (S1) task. In this setting as well, encoder-based transformer models achieve the strongest performance [31,59,60].

Beyond emrQA, several recent datasets derived from the MIMIC corpus [86] further extend discharge-summary-based Question-Answering tasks in directions more closely aligned with our Answer Prediction (S2). Unlike our approach, which targets short, schema-constrained answers, these resources focus on generating and evaluating richer, free-text responses in natural clinical language. EHRNoteQA [87] provides a benchmark for evaluating language models on complex clinical questions that may require reasoning across multiple discharge summaries. Its automatically generated question-answer pairs were refined by clinicians, and the dataset is used to assess a broad range of decoder-style transformer models in both open-ended and multiple-choice formats. MeDiSumQA [88] similarly constructs a dataset from MIMIC discharge summaries with a focus on patient-oriented question answering; its automatically generated question-answer pairs are likewise refined through manual quality control and are used to benchmark both general-purpose and biomedical language models on producing layperson-friendly responses. EHR-DS-QA [89], by contrast, adopts a fully synthetic pipeline in which question-answer pairs are generated directly from individual discharge summaries to support the development of retrieval-augmented language models for clinical information extraction.

On MIMIC [90], ArchEHR-QA shared task [91] has also been established, representing a different variant of a combination of Evidence Extraction (S1) and Answer Prediction (S2) tasks. The goal is both to predict answers to patient questions about their discharge summaries and to ensure that all answer content is explicitly grounded in the source discharge summary sentences (which allows fast validation). For simplicity, the task provides shortened discharge summaries ranging from tens to a few dozen sentences. While using third-party LLMs for end-to-end prompting might seem attractive, the underlying data are sensitive MIMIC records and cannot be shared externally. As a result, current methods rely on encoder-based models, such as BERT, to identify relevant sentences, followed by generative LLMs to produce the final patient-facing answers [92,93]. But the task still remains not fully solved: sentence-level evidence retrieval achieves *F*_1_-scores of only 50%-60%, and BLEU scores relative to clinician-authored answers reach roughly 4 to 5.

In the stroke clinical domain, previous work has examined how NLP can be applied to predictive modeling tasks [37-40]. These studies consider both classification and regression settings and use machine learning classifiers and regression models. They consistently show that incorporating unstructured clinical text from EHRs (such as discharge-related notes, histories of present illness, or imaging reports) improves predictive performance compared with models relying solely on structured data. Subsequent work focuses on extracting a broader set of stroke-related information from unstructured clinical text [41-44]. These approaches implement rule-based methods, classical machine learning models, and recurrent neural networks. For well-defined, relatively unambiguous tasks such as detecting large-vessel occlusion or silent brain infarcts, reported metrics (accuracy or area under the curve) are often high, around 95% or more. More complex or implicitly expressed attributes, such as ischemia extent, ASPECTS scores, or collateral status, typically show substantially lower performance.

More recent studies have explored the use of LLMs for stroke-related information extraction directly from unstructured clinical text [48,49]. These works formulate document-level classification tasks and schema-defined field extraction over clinical notes and discharge summaries, using prompt-based LLM inference without task-specific fine-tuning. In particular, a third-party LLM achieves 98% accuracy for coarse-grained stroke type classification, while sensitivity for ischemic stroke subtypes varies substantially, ranging from 40% to 95% depending on the subtype. Complementarily, a locally deployed LLM is shown to extract heterogeneous stroke audit variables from free-text discharge summaries with an overall item-level accuracy of 94%.

NLP methods have also proven useful in other clinical domains (eg, oncology), where BERT-based models achieved 98% accuracy for histology extraction [46] or 99.9% average accuracy for breast cancer pathology extraction, substantially outperforming rule-based methods [47], or gynecology, where only rule-based approaches were tested, reaching 83% *F*_1_-score for surgical history extraction [45].

All of the studies mentioned so far were conducted and evaluated primarily in English. However, some works have explored multilinguality in the clinical domain. Automatically expanding training data to additional languages does not necessarily yield substantial gains [31,94], and clinical pretraining of language models may not always be crucial [31]. Nevertheless, several studies specifically target information extraction from original reports in other languages, including German and French [94], Italian [95], Portuguese [96], or Dutch [97].

Our work builds on these prior NLP-based clinical information extraction studies, leveraging modern methods combining BERT-like models with LLMs. In contrast to previous work, we reveal challenges related to multilinguality, diverse reporting conventions across hospitals and countries, and varying question types within long stroke discharge summaries, providing the first cross-lingual comparison in this domain. We present the first results on a novel multilingual evidence-based question-answering dataset, achieving 89% end-to-end accuracy (77% for patient-specific questions and 95% for default items), and propose a novel task framing that decomposes the problem into evidence extraction (S1) and answer prediction (S2) to enable rapid HITL validation.

### Conclusions

This study shows that clinical Question Answering over multilingual stroke discharge summaries can be framed as an Evidence-Based Question-Answering task, with an intermediate Evidence Extraction step enabling HITL validation and allowing effective use of LLMs without relying on large computational resources and full-report context processing. While Evidence Extraction remains the main bottleneck, Answer Prediction is notably robust across languages and model sizes. Our findings indicate that the approach can meaningfully support structured data collection, particularly for well-represented question types, and can be deployed without excessive model requirements. However, generalization to new languages remains constrained by the need for target-language training data. Future work should evaluate the framework in additional clinical settings and assess its practical impact on real-world workflows.

## Supplementary material

10.2196/96347 Multimedia Appendix 1 Detailed dataset statistics for the resqEQA dataset, including report and evidence lengths and answer type distributions across six languages.

10.2196/96347 Multimedia Appendix 2 Full S1 and S2 results across all training configurations, models, and languages, including possible and impossible instances.

10.2196/96347 Multimedia Appendix 3 Performance by answer type for S1, S2, and end-to-end QA, reported across languages and split into possible and impossible instances in the T setting.

10.2196/96347 Multimedia Appendix 4 Evidence extraction error analysis showing error-type distribution, dependence on number of gold spans, qualitative span-level errors, and weak correlation with report length across languages.

10.2196/96347 Multimedia Appendix 5 Few-shot evaluation results for Answer Prediction (S2) across models and languages, showing accuracy under 0-, 1-, 5-, and 20-shot settings for all, possible, and impossible instances.

10.2196/96347 Multimedia Appendix 6 Impact of extended context length on Answer Prediction (S2) performance, showing stable accuracy across increasing report segment lengths (64–256 tokens) for all, possible, and impossible instances.

10.2196/96347 Multimedia Appendix 7 Source code for reproducing all experiments and analyses.

## Abbreviations

BERT: Bidirectional Encoder Representations from Transformers
EHR: electronic health record
EM: exact match
HITL: human in the loop
LLM: large language model
mBERT: Multilingual BERT
mmBERT: Multilingual ModernBERT
NLP: natural language processing
RAG: retrieval-augmented generation
RES-Q: Registry of Stroke Care Quality
XLMR: XLM-Roberta
