# Supplementary material for: Multilingual Evidence-Based Question-Answering for Stroke Discharge Summaries: Study of Cross-Lingual Heterogeneity in Clinical Reports
Source: J Med Internet Res. 2026 Aug 19;28:e96347. doi: 10.2196/96347 (PMC13489506; doi:10.2196/96347)
Supplement: Supplementary file 1 [file jmir-v28-e96347-s001.pdf]

Detailed Dataset Statistics

Report Lengths

	**Characters**	**Words**	**Lines**	**Paragraphs**	**mmBERT Tokens**	**Med42 Tokens**
BG	8,201	1,217	51	8	3,610.41	3,811
EL	10,742	1,417	153	23	5,534.17	5,077
EN	5,147	706	211	19	1,634.84	1,446
ES	10,877	1,592	158	2	3,701.44	3,917
PL	31,118	4,265	523	41	13,680.40	13,869
RO	26,897	4,134	1,105	28	11,408.40	10,637
**AVG**	**16,965**	**2,415**	**408**	**24**	**7,396**	**7,215**

Table 1: Average length of reports across six languages in the resqEQA dataset, expressed in characters, words, lines, paragraphs, and tokens.

Evidence Lengths

	**Characters**	**Words**
BG	23.43	3.53
EL	21.67	2.95
EN	20.30	2.80
ES	52.96	8.02
PL	48.81	6.66
RO	26.44	3.59
**AVG**	**29.44**	**4.09**

Table 2: Average length of evidences across six languages in the resqEQA dataset (aggregating all evidence spans in the evidence list), computed over possible instances only, i.e., instances with at least one evidence span in the list.

Answer Type Distribution

	Boolean	Date-Time	Enumeration	Integer	Number	String
BG	22,572	1,215	5,622	3,936	575	200
EL	23,307	1,238	4,487	2,173	590	294
EN	20,466	893	5,894	866	53	308
ES	2,447	117	594	356	41	26
PL	35,392	1,927	7,451	3,252	739	191
RO	25,710	1,196	4,507	1,934	486	149
**Total**	**129,894**	**6,586**	**28,555**	**12,517**	**2,484**	**1,168**

Table 3: Distribution of predefined form answer types in the resqEQA dataset across six languages, corresponding to the types of questions in the form: Boolean, Date-Time, Enumeration, Integer, Number, and String as an open-ended text.

	Boolean	Date-Time	Enumeration	Integer	Number	String
BG	5,038	1,103	2,615	2,586	292	45
EL	6,542	1,238	4,468	2,173	590	294
EN	4,551	886	3,986	531	53	305
ES	344	103	326	191	41	2
PL	5,878	1,716	4,690	2,141	727	185
RO	5,713	1,169	3,185	1,664	484	144
**Total**	**28,066**	**6,215**	**19,270**	**9,286**	**2,187**	**975**

Table 4: Distribution of predefined form answer types in the resqEQA dataset for possible instances across six languages, corresponding to the types of questions in the form: Boolean, Date-Time, Enumeration, Integer, Number, and String as an open-ended text.

	Boolean	Date-Time	Enumeration	Integer	Number	String
BG	17,534	112	3,007	1,350	283	155
EL	16,765	0	19	0	0	0
EN	15,915	7	1,908	335	0	3
ES	2,103	14	268	165	0	24
PL	29,514	211	2,761	1,111	12	6
RO	19,997	27	1,322	270	2	5
**Total**	**101,828**	**371**	**9,285**	**3,231**	**297**	**193**

Table 5: Distribution of predefined form answer types in the resqEQA dataset for impossible instances across six languages, corresponding to the types of questions in the form: Boolean, Date-Time, Enumeration, Integer, Number, and String as an open-ended text.
