# Supplementary material for: Multilingual Evidence-Based Question-Answering for Stroke Discharge Summaries: Study of Cross-Lingual Heterogeneity in Clinical Reports
Source: J Med Internet Res. 2026 Aug 19;28:e96347. doi: 10.2196/96347 (PMC13489506; doi:10.2196/96347)
Supplement: Supplementary file 2 [file jmir-v28-e96347-s002.pdf]

Complete Results Across Training Configurations

Training Configurations

We explore the different sets of training data and their combinations (specifically those combinations described in Table 1):

- **Target Original, T**: Original reports written in the target language. For instance, when BG is the target language under test, only the original BG training reports are used.
- **Other Original, O**: Original reports written in all other languages. For instance, when BG is the target language under test, we use the original EL, EN, PL, and RO training reports.
- **Target-to-Other Augmented, $\mathbf{T} \overset{\mathbf{aug}}{\rightarrow} \mathbf{O}$**: Reports originally written in the target language, augmented into all other languages. For instance, when BG is the target language under test, we use augmented training reports translated from the original BG reports into EL, EN, ES, PL, and RO.
- **Other-to-Target Augmented, $\mathbf{O} \overset{\mathbf{aug}}{\rightarrow} \mathbf{T}$**: Reports originally written in other languages, augmented into the target language. For instance, when BG is the target language under test, we use augmented training reports translated from original reports in EL, EN, PL, and RO into BG.
- **Other-to-Other Augmented, $\mathbf{O} \overset{\mathbf{aug}}{\rightarrow} \mathbf{O}$**: Reports originally written in other languages, augmented into other languages. For instance, when BG is the target language under test, we use augmented training reports translated from original reports in EN, EL, PL, and RO into EL, EN, ES, PL, and RO.

**T**	**O**	$\mathbf{T} \overset{\mathbf{aug}}{\rightarrow} \mathbf{O}$	$\mathbf{O} \overset{\mathbf{aug}}{\rightarrow} \mathbf{T}$	$\mathbf{O} \overset{\mathbf{aug}}{\rightarrow} \mathbf{O}$	**Description**
✓					Original reports in target language only.
✓		✓			Target-language reports with their translations to all other languages.
✓			✓		Target-language reports with augmented reports from other languages to the target language.
✓	✓				All original reports (target + other languages).
✓	✓	✓	✓	✓	All available data (original + all augmentations).
			✓		Only augmented reports from other languages into target.
	✓				Only original reports in other languages.
	✓		✓	✓	All data except original target-language reports and their augmentations.

Table 1: Combinations of training subsets explored in our experiments, combining multilingual and monolingual data from original and augmented reports. Checkmarks indicate the inclusion of specific data subset in each configuration: Target Original (T), Other Original (O), Target-to-Other Augmented ($T \overset{aug}{\rightarrow} O$), Other-to-Target Augmented ($O \overset{aug}{\rightarrow} T$), and Other-to-Other Augmented ($O \overset{aug}{\rightarrow} O$).

Evidence Extraction (S1)

Table 2 reports the results of the XLMR, mBERT, and mmBERT models across all possible combinations of training data variants, including both original and augmented reports. We report F1 and EM scores computed over all test instances. Table 6 presents the results computed only on possible instances, while Table 7 reports the results on the remaining instances, i.e., impossible instances.

**Evidence Extraction F1**												
	**T**	**O**	**T $\xrightarrow{aug}$ O**	**O $\xrightarrow{aug}$ T**	**O $\xrightarrow{aug}$ O**	**BG**	**EL**	**EN**	**PL**	**RO**	**AVG**	***ES***
XLMR	✓					85.85	86.62	75.85	**81.18**	**83.75**	82.65	–
	✓		✓			**86.73**	**87.23**	**80.09**	80.51	83.23	**83.56**	–
	✓			✓		83.32	81.43	78.45	75.23	77.62	79.21	–
	✓	✓				83.71	82.39	78.34	74.93	78.30	79.53	–
	✓	✓	✓	✓	✓	84.95	85.23	78.17	76.16	81.07	81.12	–
				✓		65.32	48.58	63.04	68.74	62.06	61.55	**71.55**
		✓				66.91	51.77	63.41	68.19	63.43	62.74	68.82
		✓		✓	✓	68.93	53.01	63.27	69.35	66.37	64.19	71.05
mBERT	✓					88.86	**87.61**	**79.46**	**80.71**	**82.52**	**83.83**	–
	✓		✓			**89.09**	85.88	79.78	68.58	78.73	80.41	–
	✓			✓		84.22	72.45	77.83	73.22	75.31	76.61	–
	✓	✓				85.25	78.20	76.39	74.38	74.48	77.74	
	✓	✓	✓	✓	✓	85.83	79.99	77.01	74.56	77.14	78.91	–
				✓		65.27	52.91	63.02	67.46	63.29	62.39	71.41
		✓				61.37	54.80	63.65	67.88	62.95	62.13	71.91
		✓		✓	✓	67.97	53.66	63.18	67.31	64.75	63.38	**72.23**
mmBERT	✓					**90.53**	**90.12**	**80.72**	**81.66**	80.80	**84.77**	–
	✓		✓			89.79	87.71	79.54	81.44	**81.21**	83.94	–
	✓			✓		87.83	85.49	79.66	75.58	75.70	80.85	–
	✓	✓				87.45	83.90	79.97	75.24	76.71	80.65	-
	✓	✓	✓	✓	✓	87.95	84.92	78.22	76.57	77.53	81.03	-
				✓		56.56	52.40	58.81	66.25	62.21	59.25	70.39
		✓				66.76	55.16	63.48	66.50	63.38	63.06	66.81
		✓		✓	✓	69.89	55.63	63.25	69.33	66.59	64.94	**72.78**
**Evidence Extraction EM**												
XLMR	✓					80.54	79.95	69.67	74.61	**77.17**	76.39	–
	✓		✓			**81.45**	**80.68**	**74.00**	**74.63**	77.10	**77.57**	–
	✓			✓		78.66	74.35	72.74	70.20	72.53	73.69	–
	✓	✓				78.88	75.28	72.59	69.61	72.18	73.71	–
	✓	✓	✓	✓	✓	79.66	77.37	72.21	70.06	74.65	74.79	–
				✓		64.27	48.34	62.88	68.00	60.27	60.75	**70.54**
		✓				66.15	48.92	63.12	66.78	62.47	61.49	66.88
		✓		✓	✓	65.39	49.70	63.14	66.68	62.74	61.53	68.39
mBERT	✓					**83.42**	**80.75**	73.07	**74.32**	**76.21**	**77.55**	–
	✓		✓			82.86	78.26	**73.29**	68.58	73.02	75.20	–
	✓			✓		80.01	66.43	71.55	70.55	70.55	71.82	–
	✓	✓				80.91	71.39	72.25	70.40	70.82	73.15	–
	✓	✓	✓	✓	✓	81.16	73.91	72.14	71.12	72.50	74.17	–
				✓		64.76	52.53	63.01	67.21	61.26	61.75	70.59
		✓				60.48	52.39	63.28	66.95	62.63	61.15	**71.13**
		✓		✓	✓	66.73	52.82	63.12	65.56	62.68	62.18	70.06
mmBERT	✓					**85.05**	**83.46**	**74.75**	**75.29**	**74.26**	**78.56**	–
	✓		✓			84.40	80.28	73.34	75.16	74.14	77.46	–
	✓			✓		82.34	77.46	72.96	69.59	69.03	74.28	–
	✓	✓				81.46	75.41	73.58	71.03	69.98	74.29	-
	✓	✓	✓	✓	✓	83.17	77.65	72.94	71.96	71.93	75.53	-
				✓		55.45	52.40	58.45	64.72	59.60	58.13	69.11
		✓				66.10	53.49	62.92	64.62	61.32	61.69	63.50
		✓		✓	✓	67.51	53.85	63.08	66.44	62.62	62.70	**70.57**

Table 2: Evidence extraction (S1) performance (F1 and EM) across the XLMR, mBERT, and mmBERT models under different training configurations combining monolingual and multilingual data with augmented reports.

Evidence Extraction F1												
	**T**	**O**	**T** $\overset{\text{aug}}{\rightarrow}$ **O**	**O** $\overset{\text{aug}}{\rightarrow}$ **T**	**O** $\overset{\text{aug}}{\rightarrow}$ **O**	**BG**	**EL**	**EN**	**PL**	**RO**	**AVG**	***ES***
XLMR	✓					64.38	73.53	46.77	**44.44**	**61.81**	58.18	–
	✓		✓			**66.93**	**75.63**	**52.49**	41.90	60.21	**59.43**	–
	✓			✓		55.51	64.09	47.20	25.19	43.29	47.06	–
	✓	✓				56.36	65.16	47.95	26.72	47.37	48.71	-
	✓	✓	✓	✓	✓	61.48	71.03	47.98	32.46	54.41	53.47	-
				✓		4.27	0.59	1.02	3.23	8.46	3.51	4.59
		✓				4.11	7.50	1.58	5.23	4.54	4.59	9.09
		✓		✓	✓	17.38	10.31	0.76	12.26	18.06	11.75	**14.24**
mBERT	✓					72.79	**76.19**	51.96	**42.63**	**58.60**	**60.43**	–
	✓		✓			**75.16**	72.69	**52.58**	0.00	47.74	49.63	–
	✓			✓		55.55	44.86	48.85	17.17	37.93	40.87	–
	✓	✓				59.12	56.29	39.47	21.80	34.38	42.21	-
	✓	✓	✓	✓	✓	61.20	59.07	41.39	20.90	41.98	44.91	-
				✓		2.09	1.13	0.03	1.01	7.46	2.34	3.80
		✓				3.84	6.14	2.57	3.67	1.62	3.57	4.18
		✓		✓	✓	6.97	2.80	0.67	7.19	8.88	5.30	**11.16**
mmBERT	✓					**75.92**	**80.51**	**52.16**	**46.93**	53.63	**61.83**	–
	✓		✓			74.27	75.82	50.86	45.38	**55.89**	60.45	–
	✓			✓		68.89	71.93	50.42	32.65	45.82	53.94	–
	✓	✓				68.95	70.33	52.11	26.88	45.08	52.67	-
	✓	✓	✓	✓	✓	67.15	69.42	45.84	29.37	44.45	51.24	-
				✓		4.75	0.00	1.37	6.40	11.10	4.72	6.23
		✓				3.46	6.00	2.86	8.68	10.19	6.24	**14.65**
		✓		✓	✓	13.89	7.00	1.10	13.43	16.93	10.47	11.55
**Evidence Extraction EM**												
XLMR	✓					49.54	58.70	30.18	**23.56**	**44.34**	41.27	–
	✓		✓			**52.81**	**61.22**	**36.76**	23.33	**44.34**	**43.69**	–
	✓			✓		41.72	48.30	32.21	9.17	29.41	32.16	–
	✓	✓				42.14	49.48	32.61	9.81	30.66	32.94	-
	✓	✓	✓	✓	✓	47.14	53.76	32.16	13.08	37.52	36.73	-
				✓		1.20	0.10	0.59	0.88	3.69	1.29	0.99
		✓				1.90	1.47	0.79	0.72	1.97	1.37	2.18
		✓		✓	✓	7.00	3.24	0.40	3.87	8.39	4.58	**4.77**
mBERT	✓					57.28	**61.67**	35.18	**22.37**	**41.93**	**43.69**	–
	✓		✓			**57.99**	56.47	**35.82**	0.00	32.83	36.62	–
	✓			✓		43.13	32.01	32.26	8.61	25.00	28.20	–
	✓	✓				46.32	41.60	28.70	9.01	24.64	30.05	-
	✓	✓	✓	✓	✓	48.10	46.11	28.70	9.93	29.78	32.52	-
				✓		0.58	0.33	0.00	0.24	2.13	0.65	0.89
		✓				1.20	1.08	1.58	0.72	0.76	1.07	1.39
		✓		✓	✓	3.27	1.05	0.49	1.71	3.25	1.96	**3.48**
mmBERT	✓					**61.30**	**65.93**	**36.62**	**25.92**	36.32	**45.21**	–
	✓		✓			59.81	59.68	34.59	24.40	**37.64**	43.22	–
	✓			✓		53.56	54.55	33.00	13.32	27.97	36.48	–
	✓	✓				52.11	52.13	35.33	13.36	27.29	36.04	-
	✓	✓	✓	✓	✓	54.22	53.53	32.01	14.43	29.25	36.69	-
				✓		1.53	0.00	0.40	1.52	4.01	1.49	1.69
		✓				1.49	2.49	1.24	2.71	4.61	2.51	2.88
		✓		✓	✓	6.87	3.24	0.64	4.27	6.26	4.26	**3.67**

Table 3: Evidence extraction (S1) performance (F1 and EM) across the XLMR, mBERT, and mmBERT models under different training configurations combining monolingual and multilingual data with augmented reports for possible instances.

**Evidence Extraction F1**												
	**T**	**O**	**T $\xrightarrow{\text{aug}}$ O**	**O $\xrightarrow{\text{aug}}$ T**	**O $\xrightarrow{\text{aug}}$ O**	**BG**	**EL**	**EN**	**PL**	**RO**	**AVG**	***ES***
XLMR	✓					96.80	**98.51**	92.92	98.01	96.88	96.62	–
	✓		✓			96.82	97.77	96.28	**98.19**	97.00	97.21	–
	✓			✓		97.49	97.18	96.78	98.15	98.15	**97.55**	–
	✓	✓				97.64	98.04	96.17	97.02	96.81	97.14	-
	✓	✓	✓	✓	✓	96.90	98.13	95.88	96.18	97.02	96.82	-
				✓		96.42	92.16	99.42	98.76	94.12	96.17	**97.75**
		✓				**98.90**	91.98	99.68	97.04	**98.66**	97.25	92.19
		✓		✓	✓	95.19	91.80	**99.94**	95.51	95.27	95.54	93.28
mBERT	✓					97.05	97.98	95.59	98.15	96.83	97.12	–
	✓		✓			96.18	97.86	95.73	**100.00**	97.26	97.41	–
	✓			✓		98.82	97.51	94.83	98.90	97.67	97.55	–
	✓	✓				98.57	98.10	98.06	98.47	98.46	98.33	-
	✓	✓	✓	✓	✓	98.38	98.99	97.91	99.14	98.18	**98.52**	-
				✓		97.45	**99.94**	**99.97**	97.90	96.69	98.39	97.86
		✓				90.68	98.99	99.48	97.30	**99.64**	97.22	**98.41**
		✓		✓	✓	**99.05**	99.85	99.85	94.85	98.18	98.36	96.11
mmBERT	✓					97.98	98.84	97.47	97.57	97.29	97.83	–
	✓		✓			97.70	98.51	96.37	97.95	96.35	97.38	–
	✓			✓		97.47	97.80	96.81	95.25	93.57	96.18	–
	✓	✓				96.88	96.23	96.31	97.39	95.63	96.49	-
	✓	✓	✓	✓	✓	98.55	98.99	97.21	**98.19**	97.31	**98.05**	-
				✓		82.94	**100.00**	92.51	93.68	92.77	92.38	95.49
		✓				**99.01**	99.82	99.04	92.99	95.20	97.21	87.22
		✓		✓	✓	98.42	99.79	**99.71**	94.94	**96.30**	97.83	**96.74**
**Evidence Extraction EM**												
XLMR	✓					96.80	**98.51**	92.92	98.01	96.88	96.62	–
	✓		✓			96.82	97.77	96.28	**98.19**	97.00	97.21	–
	✓			✓		97.49	97.18	96.78	98.15	98.15	**97.55**	–
	✓	✓				97.64	98.04	96.17	97.02	96.81	97.14	-
	✓	✓	✓	✓	✓	96.90	98.13	95.88	96.18	97.02	96.82	-
				✓		96.42	92.16	99.42	98.76	94.12	96.17	**97.75**
		✓				**98.90**	91.98	99.68	97.04	**98.66**	97.25	92.19
		✓		✓	✓	95.19	91.80	**99.94**	95.51	95.27	95.54	93.28
mBERT	✓					97.05	97.98	95.59	98.15	96.83	97.12	–
	✓		✓			96.18	97.86	95.73	**100.00**	97.26	97.41	–
	✓			✓		98.82	97.51	94.83	98.90	97.67	97.55	–
	✓	✓				98.57	98.10	98.06	98.47	98.46	98.33	-
	✓	✓	✓	✓	✓	98.38	98.99	97.91	99.14	98.18	**98.52**	-
				✓		97.45	**99.94**	**99.97**	97.90	96.69	98.39	97.86
		✓				90.68	98.99	99.48	97.30	**99.64**	97.22	**98.41**
		✓		✓	✓	**99.05**	99.85	99.85	94.85	98.18	98.36	96.11
mmBERT	✓					97.98	98.84	97.47	97.57	97.29	97.83	–
	✓		✓			97.70	98.51	96.37	97.95	96.35	97.38	–
	✓			✓		97.47	97.80	96.81	95.25	93.57	96.18	–
	✓	✓				96.88	96.23	96.31	97.39	95.63	96.49	-
	✓	✓	✓	✓	✓	98.55	98.99	97.21	**98.19**	97.31	**98.05**	-
				✓		82.94	**100.00**	92.51	93.68	92.77	92.38	95.49
		✓				**99.01**	99.82	99.04	92.99	95.20	97.21	87.22
		✓		✓	✓	98.42	99.79	**99.71**	94.94	**96.30**	97.83	**96.74**

Table 4: Evidence extraction (S1) performance (F1 and EM) across the XLMR, mBERT, and mmBERT models under different training configurations combining monolingual and multilingual data with augmented reports for impossible instances.

Clinical vs. Multilingual Models on English Reports

Table 5 compares English clinical models against multilingual non-clinical models on the English test set of Evidence Extraction task. Tables 6 and 7 provide a more detailed analysis by reporting results separately for possible and impossible instances, respectively.

Evidence Extraction F1 - Multilingual vs. Clinical								
**T**	**O**	**T $\overset{aug}{\to}$ O**	**O $\overset{aug}{\to}$ T**	**O $\overset{aug}{\to}$ O**	**mBERT**	**ClinicalBERT**	**mmBERT**	**ClinicalModernBERT**
✓					79.46	69.36	**80.72**	68.57
✓			✓		77.83	69.26	**79.66**	75.13
			✓		63.02	**63.03**	58.81	62.47
**Evidence Extraction EM - Multilingual vs. Clinical**								
**T**	**O**	**T $\overset{aug}{\to}$ O**	**O $\overset{aug}{\to}$ T**	**O $\overset{aug}{\to}$ O**	**mBERT**	**ClinicalBERT**	**mmBERT**	**ClinicalModernBERT**
✓					73.07	66.76	**74.75**	66.25
✓			✓		71.55	66.70	**72.96**	70.12
			✓		63.01	**63.03**	58.45	62.46

Table 5: Evidence Extraction (S1) performance (F1 and EM) on English test reports when training on the combinations of T and $O \overset{aug}{\to} T$ subsets, comparing the clinical models ClinicalBERT and ClinicalModernBERT with their multilingual non-clinical counterparts mBERT and mmBERT.

Evidence Extraction F1 - Multilingual vs. Clinical								
**T**	**O**	**T $\overset{aug}{\to}$ O**	**O $\overset{aug}{\to}$ T**	**O $\overset{aug}{\to}$ O**	**mBERT**	**ClinicalBERT**	**mmBERT**	**ClinicalModernBERT**
✓					51.96	18.56	**52.16**	17.53
✓			✓		48.85	19.27	**50.42**	37.57
			✓		0.03	0.00	**1.37**	0.03
**Evidence Extraction EM - Multilingual vs. Clinical**								
**T**	**O**	**T $\overset{aug}{\to}$ O**	**O $\overset{aug}{\to}$ T**	**O $\overset{aug}{\to}$ O**	**mBERT**	**ClinicalBERT**	**mmBERT**	**ClinicalModernBERT**
✓					35.18	11.63	**36.62**	11.38
✓			✓		32.26	12.37	**33.00**	24.25
			✓		0.00	0.00	**0.40**	0.00

Table 6: Evidence Extraction (S1) performance (F1 and EM) on English test reports for possible instances when training on the combinations of T and $O \overset{aug}{\to} T$ subsets, comparing the clinical models ClinicalBERT and ClinicalModernBERT with their multilingual non-clinical counterparts mBERT and mmBERT.

Evidence Extraction F1 - Multilingual vs. Clinical								
**T**	**O**	**T $\overset{aug}{\to}$ O**	**O $\overset{aug}{\to}$ T**	**O $\overset{aug}{\to}$ O**	**mBERT**	**ClinicalBERT**	**mmBERT**	**ClinicalModernBERT**
✓					95.59	**99.16**	97.47	98.52
✓			✓		94.83	**98.58**	96.81	97.16
			✓		99.97	**100.00**	92.51	99.10
**Evidence Extraction EM - Multilingual vs. Clinical**								
**T**	**O**	**T $\overset{aug}{\to}$ O**	**O $\overset{aug}{\to}$ T**	**O $\overset{aug}{\to}$ O**	**mBERT**	**ClinicalBERT**	**mmBERT**	**ClinicalModernBERT**
✓					95.59	**99.16**	97.47	98.52
✓			✓		94.83	**98.58**	96.81	97.16
			✓		99.97	**100.00**	92.51	99.10

Table 7: Evidence Extraction (S1) performance (F1 and EM) on English test reports for impossible instances when training on the combinations of T and $O \overset{aug}{\to} T$ subsets, comparing the clinical models ClinicalBERT and ClinicalModernBERT with their multilingual non-clinical counterparts mBERT and mmBERT.

Answer Prediction (S2)

Table 8 presents the results for all training data configurations in the Answer Prediction task, comparing four general-purpose generative models and their four clinical counterparts. Results for possible instances are reported separately in Table 9, while results for impossible instances are provided in Table 10.

	T	O	T $\xrightarrow{aug}$ O	O $\xrightarrow{aug}$ T	O $\xrightarrow{aug}$ O	BG	EL	EN	PL	RO	AVG	*ES*
MediPhi	✓					92.79	97.03	93.54	92.02	96.12	94.30	–
	✓		✓			**93.17**	**97.32**	**94.49**	**92.31**	**97.03**	**94.86**	–
	✓			✓		92.57	97.31	94.37	92.14	96.85	94.65	–
	✓	✓				92.90	97.21	93.45	91.47	96.83	94.37	–
	✓	✓	✓	✓	✓	92.83	97.43	93.08	91.87	96.98	94.44	–
				✓		69.24	58.38	82.80	84.28	83.51	75.64	50.10
		✓				85.03	91.91	85.38	82.77	92.39	87.49	85.26
		✓		✓	✓	84.37	93.06	84.12	84.24	92.79	87.72	**86.34**
Phi3.5 Mini	✓					92.81	97.25	93.85	91.77	96.40	94.41	–
	✓		✓			**93.28**	**97.53**	**94.46**	**92.33**	**96.89**	**94.90**	–
	✓			✓		92.88	97.48	94.00	92.14	96.76	94.65	–
	✓	✓				92.88	97.39	92.97	91.58	96.76	94.31	–
	✓	✓	✓	✓	✓	92.88	97.28	93.30	91.87	96.88	94.44	–
				✓		83.84	90.35	81.19	84.06	91.57	86.20	66.49
		✓				84.48	94.23	83.85	81.41	92.75	87.34	85.90
		✓		✓	✓	84.65	94.55	85.33	83.84	92.36	88.14	**86.57**
MedGemma	✓					92.54	97.48	93.56	92.33	96.86	94.55	–
	✓		✓			**93.31**	**97.95**	**94.57**	**92.62**	96.85	**95.06**	–
	✓			✓		92.60	97.79	93.71	92.55	96.77	94.68	–
	✓	✓				93.14	97.65	93.52	91.83	**96.91**	94.61	–
	✓	✓	✓	✓	✓	93.17	97.63	92.35	92.41	96.77	94.47	–
				✓		68.03	47.32	60.76	80.06	81.98	67.63	56.10
		✓				86.56	95.32	87.72	85.42	91.96	89.40	**86.57**
		✓		✓	✓	86.30	94.94	86.00	85.53	92.58	89.07	85.09
Gemma3	✓					92.55	**98.01**	93.72	91.87	96.61	94.55	–
	✓		✓			**93.24**	86.82	**94.62**	92.31	**96.98**	92.79	–
	✓			✓		92.15	97.40	94.18	92.42	96.61	94.55	–
	✓	✓				92.42	97.65	93.14	91.36	96.80	94.27	–
	✓	✓	✓	✓	✓	93.07	97.90	92.57	**92.53**	**96.98**	**94.61**	–
				✓		83.89	92.05	77.08	83.88	89.31	85.24	83.27
		✓				86.23	95.33	87.14	84.47	91.77	88.99	86.51
		✓		✓	✓	85.70	95.21	84.60	85.13	93.48	88.82	**86.65**
LLaMA3	✓					92.95	97.79	94.49	**92.71**	97.03	94.99	–
	✓		✓			**93.32**	97.73	**94.71**	92.72	96.73	**95.04**	–
	✓			✓		93.07	**97.85**	94.09	92.63	96.92	94.91	–
	✓	✓				93.18	97.56	94.31	91.73	96.95	94.75	–
	✓	✓	✓	✓	✓	92.99	97.81	92.77	92.32	**97.04**	94.59	–
				✓		72.29	83.39	75.59	82.46	89.56	80.66	84.31
		✓				85.36	95.74	87.21	82.57	92.39	88.65	84.14
		✓		✓	✓	85.47	94.01	84.49	85.45	93.05	88.49	**87.01**
BioMistral	✓					93.09	97.68	94.80	92.42	97.13	95.02	–
	✓		✓			**93.31**	97.68	94.73	92.61	97.12	95.09	–
	✓			✓		93.21	**97.84**	**94.90**	**92.70**	97.00	**95.13**	–
	✓	✓				93.24	97.82	93.30	92.27	**97.16**	94.76	–
	✓	✓	✓	✓	✓	93.27	97.76	93.07	92.32	97.00	94.68	–
				✓		72.08	79.74	77.50	85.44	84.67	79.88	80.56
		✓				86.37	92.67	86.55	85.67	93.74	89.00	**86.85**
		✓		✓	✓	85.63	93.74	85.53	85.40	93.42	88.74	86.26
Mistral	✓					93.27	97.76	94.57	92.60	**97.16**	95.07	–
	✓		✓			93.34	97.51	**94.58**	92.41	97.00	94.97	–
	✓			✓		**93.45**	**97.87**	**94.58**	**92.81**	97.04	**95.15**	–
	✓	✓				93.21	97.74	93.34	92.26	97.00	94.71	–
	✓	✓	✓	✓	✓	92.95	97.63	93.18	92.01	96.94	94.54	–
				✓		59.49	60.90	57.28	80.93	86.02	68.92	51.08
		✓				85.14	93.12	86.57	84.45	93.51	88.56	**86.26**
		✓		✓	✓	84.93	93.59	84.49	84.97	93.20	88.23	86.23
Med42	✓					93.04	97.82	94.88	**92.75**	96.91	95.08	–
	✓		✓			93.36	**97.85**	**94.97**	92.50	96.92	**95.12**	–
	✓			✓		**93.39**	97.76	94.51	92.72	**97.21**	**95.12**	–
	✓	✓				93.06	97.84	94.04	92.06	97.16	94.83	–
	✓	✓	✓	✓	✓	92.99	97.76	93.34	92.19	97.04	94.66	–
				✓		78.35	88.81	80.83	85.79	92.52	85.26	83.94
		✓				86.45	95.36	87.27	84.73	93.38	89.44	**86.40**
		✓		✓	✓	84.97	93.96	84.36	85.39	94.47	88.63	86.37

Table 8: Answer Prediction (S2) results for models trained with combinations of various configurations.

	**T**	**O**	**T $\overset{aug}{\rightarrow}$ O**	**O $\overset{aug}{\rightarrow}$ T**	**O $\overset{aug}{\rightarrow}$ O**	**BG**	**EL**	**EN**	**PL**	**RO**	**AVG**	***ES***
MediPhi	✓					93.63	93.85	88.67	88.88	92.94	91.59	–
	✓		✓			**94.91**	94.41	**90.70**	89.75	94.58	92.87	–
	✓			✓		94.08	94.41	90.35	89.27	94.42	92.51	–
	✓	✓				94.29	94.24	90.10	89.43	94.18	92.45	-
	✓	✓	✓	✓	✓	94.54	**94.70**	90.05	**90.47**	**94.82**	**92.92**	-
				✓		86.71	83.29	76.05	79.98	86.60	82.53	82.22
		✓				86.96	83.09	80.26	79.74	88.24	83.66	83.71
		✓		✓	✓	84.85	85.51	74.96	81.66	87.32	82.86	**87.69**
Phi3.5 Mini	✓					93.87	94.28	89.31	87.92	93.50	91.78	–
	✓		✓			**95.07**	**94.87**	90.60	90.07	94.46	93.01	–
	✓			✓		94.41	**94.87**	90.20	89.19	94.30	92.60	–
	✓	✓				94.16	94.64	89.96	88.92	94.02	92.34	-
	✓	✓	✓	✓	✓	94.62	94.38	**91.04**	**90.43**	**94.66**	**93.03**	-
				✓		84.02	82.28	71.50	79.74	85.11	80.53	84.31
		✓				85.97	88.00	76.50	79.74	89.25	83.89	84.41
		✓		✓	✓	84.35	88.65	77.78	80.54	87.52	83.77	**85.30**
MedGemma	✓					93.79	94.77	89.81	89.75	94.26	92.48	–
	✓		✓			**95.32**	**95.72**	91.34	90.79	94.50	**93.53**	–
	✓			✓		94.04	95.36	90.30	90.55	94.42	92.93	–
	✓	✓				94.50	95.16	**91.44**	90.03	**94.54**	93.13	-
	✓	✓	✓	✓	✓	95.16	95.13	90.80	**91.03**	94.46	93.31	-
				✓		87.13	86.72	78.03	83.49	85.63	84.20	85.80
		✓				88.37	90.26	84.56	84.49	86.76	86.89	**87.49**
		✓		✓	✓	87.04	89.44	79.61	85.49	87.52	85.82	84.61
Gemma3	✓					94.12	**95.88**	90.75	89.95	94.10	92.96	–
	✓		✓			**95.32**	95.42	90.99	89.83	94.82	93.28	–
	✓			✓		93.75	94.57	90.75	90.27	94.30	92.73	–
	✓	✓				94.58	95.36	90.50	90.43	94.58	93.09	-
	✓	✓	✓	✓	✓	95.20	95.65	**91.29**	**91.03**	**94.98**	**93.63**	-
				✓		87.38	85.68	77.14	81.74	84.51	83.29	85.10
		✓				88.37	90.29	83.62	82.81	86.60	86.34	85.20
		✓		✓	✓	86.75	89.99	75.66	84.21	90.37	85.40	**87.49**
LLaMA3	✓					94.87	95.39	91.14	**91.35**	94.82	93.51	–
	✓		✓			95.16	95.26	91.39	91.19	94.22	93.44	–
	✓			✓		95.16	**95.49**	91.34	90.95	94.78	**93.54**	–
	✓	✓				**95.28**	94.96	**92.23**	90.91	94.78	93.63	-
	✓	✓	✓	✓	✓	94.91	**95.49**	90.95	91.11	**95.06**	93.50	-
				✓		88.08	88.46	79.22	81.58	89.49	85.36	85.60
		✓				88.08	91.17	83.62	82.54	87.32	86.55	86.99
		✓		✓	✓	88.04	87.48	75.36	84.85	89.93	85.13	**88.68**
BioMistral	✓					94.99	95.16	91.54	90.31	94.90	93.38	–
	✓		✓			95.41	95.19	91.34	90.71	95.06	93.54	–
	✓			✓		95.16	95.45	**91.74**	90.83	94.82	93.60	–
	✓	✓				**95.45**	**95.52**	91.14	90.91	**95.10**	**93.62**	-
	✓	✓	✓	✓	✓	94.91	95.39	90.70	**91.47**	94.86	93.47	-
				✓		84.64	84.43	79.07	83.45	91.25	84.57	84.51
		✓				88.49	84.70	81.54	85.65	90.61	86.20	85.40
		✓		✓	✓	86.75	86.95	78.48	85.73	90.49	85.68	**85.90**
Mistral	✓					95.07	95.36	90.90	90.91	**95.06**	93.46	–
	✓		✓			95.12	94.80	90.90	90.35	94.82	93.20	–
	✓			✓		**95.36**	**95.52**	**90.95**	**91.39**	94.90	**93.62**	–
	✓	✓				94.95	95.36	90.90	91.27	95.02	93.50	-
	✓	✓	✓	✓	✓	95.07	95.13	90.85	90.67	94.74	93.29	-
				✓		84.52	82.41	77.78	82.54	89.41	83.33	86.10
		✓				87.33	85.64	81.59	83.69	90.05	85.66	85.00
		✓		✓	✓	85.89	86.59	75.90	85.01	89.97	84.67	**86.89**
Med42	✓					94.95	95.45	**92.03**	91.27	94.78	93.70	–
	✓		✓			**95.36**	95.55	91.93	90.67	94.66	93.64	–
	✓			✓		95.28	95.32	91.39	**91.55**	**95.43**	**93.79**	–
	✓	✓				95.24	**95.59**	91.29	91.51	95.22	93.77	-
	✓	✓	✓	✓	✓	95.12	95.39	91.24	90.83	95.18	93.55	-
				✓		86.47	88.78	77.19	82.34	87.68	84.49	84.51
		✓				88.04	90.35	83.23	85.09	90.25	87.39	85.50
		✓		✓	✓	86.18	87.41	75.61	85.61	91.45	85.25	**87.09**

Table 9: Answer Prediction (S2) results for models trained with combinations of various configurations for possible instances.

	T	O	T $\xrightarrow{aug}$ O	O $\xrightarrow{aug}$ T	O $\xrightarrow{aug}$ O	BG	EL	EN	PL	RO	AVG	*ES*
MediPhi	✓					**92.37**	99.91	96.40	93.46	98.03	96.03	–
	✓		✓			92.28	**99.97**	**96.72**	**93.48**	**98.49**	**96.19**	–
	✓			✓		91.80	99.94	**96.72**	93.46	98.30	96.04	–
	✓	✓				92.20	99.91	95.41	92.40	98.42	95.67	-
	✓	✓	✓	✓	✓	91.97	99.91	94.86	92.51	98.27	95.50	-
				✓		60.34	35.76	86.76	86.24	81.66	70.15	37.53
		✓				84.04	99.91	88.39	84.16	94.86	90.27	**85.86**
		✓		✓	✓	84.12	99.91	89.49	85.42	96.06	91.00	85.82
Phi3.5 Mini	✓					92.26	**99.94**	96.52	**93.53**	98.13	96.08	–
	✓		✓			**92.37**	**99.94**	**96.72**	93.37	98.34	**96.15**	–
	✓			✓		92.09	98.85	96.23	93.50	98.22	95.98	–
	✓	✓				92.22	99.88	94.75	92.80	98.39	95.61	-
	✓	✓	✓	✓	✓	91.99	99.91	94.63	92.53	98.20	95.45	-
				✓		83.74	97.68	86.88	86.04	95.44	89.96	59.52
		✓				83.72	99.88	88.16	82.17	94.84	89.75	86.48
		✓		✓	✓	84.80	99.91	89.75	85.35	**95.25**	91.01	**87.06**
MedGemma	✓					91.90	99.94	95.76	**93.51**	**98.42**	95.91	–
	✓		✓			92.28	99.97	**96.46**	93.46	98.25	**96.08**	–
	✓			✓		91.86	**100.00**	95.70	93.46	98.18	95.84	–
	✓	✓				**92.45**	99.91	94.75	92.66	98.32	95.62	-
	✓	✓	✓	✓	✓	92.16	99.91	93.27	93.04	98.15	95.30	-
				✓		58.30	11.52	50.62	78.48	79.79	55.74	44.48
		✓				85.64	99.91	89.58	85.84	95.08	91.21	**86.21**
		✓		✓	✓	85.92	99.94	89.75	85.55	95.61	91.35	85.28
Gemma3	✓					91.76	99.94	95.47	92.75	98.10	95.60	–
	✓		✓			**92.18**	79.00	**96.75**	**93.44**	**98.27**	91.93	–
	✓			✓		91.33	**99.97**	96.20	93.41	97.98	**95.78**	–
	✓	✓				91.31	99.73	94.69	91.78	98.13	95.13	-
	✓	✓	✓	✓	✓	91.99	99.94	93.32	93.22	98.18	95.33	-
				✓		82.12	97.83	77.04	84.86	92.17	86.80	82.56
		✓				85.14	99.91	89.20	85.22	94.86	90.87	**87.02**
		✓		✓	✓	85.16	99.94	89.84	85.55	95.34	91.17	86.32
LLaMA3	✓					91.97	99.97	96.46	93.33	**98.34**	96.01	–
	✓		✓			**92.39**	99.97	**96.66**	**93.42**	98.22	**96.13**	–
	✓			✓		92.01	**100.00**	95.70	93.41	98.20	95.86	–
	✓	✓				92.11	99.91	95.53	92.11	98.25	95.58	-
	✓	✓	✓	✓	✓	92.01	99.91	93.85	92.88	98.22	95.37	-
				✓		64.24	78.79	73.47	82.86	89.61	77.80	83.80
		✓				83.98	99.88	89.32	82.59	95.42	90.24	83.02
		✓		✓	✓	84.17	99.94	89.84	85.73	94.91	90.92	**86.36**
BioMistral	✓					92.11	99.97	96.72	93.39	**98.46**	96.13	–
	✓		✓			92.24	99.94	96.72	93.48	98.34	96.14	–
	✓			✓		92.22	**100.00**	**96.75**	**93.55**	98.30	**96.16**	–
	✓	✓				92.11	99.91	94.57	92.89	98.39	95.58	-
	✓	✓	✓	✓	✓	**92.43**	99.91	94.46	92.71	98.27	95.56	-
				✓		65.68	75.47	76.57	86.35	80.72	76.96	79.02
		✓				85.28	99.91	89.49	85.68	95.61	91.19	**87.41**
		✓		✓	✓	85.05	99.91	89.67	85.26	95.18	91.01	86.40
Mistral	✓					92.35	99.94	96.72	93.37	**98.42**	96.16	–
	✓		✓			92.43	99.97	**96.75**	93.35	98.30	96.16	–
	✓			✓		**92.47**	**100.00**	96.72	**93.46**	98.32	**96.19**	–
	✓	✓				92.33	99.91	94.78	92.71	98.18	95.58	-
	✓	✓	✓	✓	✓	91.86	99.91	94.54	92.62	98.25	95.44	-
				✓		46.74	41.37	45.25	80.20	83.99	59.51	37.37
		✓				84.02	99.91	89.49	84.80	95.58	90.76	**86.75**
		✓		✓	✓	84.44	99.94	89.52	84.95	95.13	90.80	85.98
Med42	✓					92.07	**99.97**	96.55	**93.42**	98.18	96.04	–
	✓		✓			92.35	99.94	**96.75**	93.33	98.27	**96.13**	–
	✓			✓		**92.43**	**99.97**	96.34	93.26	98.27	96.05	–
	✓	✓				91.95	99.88	95.65	92.31	98.32	95.62	-
	✓	✓	✓	✓	✓	91.90	99.91	94.57	92.82	98.15	95.47	-
				✓		74.21	88.83	82.96	87.38	95.42	85.76	83.72
		✓				85.64	99.91	89.64	84.56	95.25	91.00	**86.75**
		✓		✓	✓	84.36	99.91	89.49	85.29	**96.28**	91.07	86.09

Table 10: Answer Prediction (S2) results for models trained with combinations of various configurations for impossible instances.
