# Supplementary material for: Multilingual Evidence-Based Question-Answering for Stroke Discharge Summaries: Study of Cross-Lingual Heterogeneity in Clinical Reports
Source: J Med Internet Res. 2026 Aug 19;28:e96347. doi: 10.2196/96347 (PMC13489506; doi:10.2196/96347)
Supplement: Supplementary file 3 [file jmir-v28-e96347-s003.pdf]

Results by Answer Type

Evidence Extraction (S1)

Table 1 reports F1 and EM scores broken down by final answer type for mmBERT evidences predictions over all test instances. Table 2 reports scores computed only over possible instances, while Table 3 reports scores computed over the remaining instances, i.e., impossible instances.

**Evidence Extraction F1 - mmBERT**						
**Type**	**BG**	**EL**	**EN**	**PL**	**RO**	**AVG**
Boolean	92.39	92.81	86.68	87.21	87.03	88.39
Date-Time	81.92	84.10	57.55	56.72	28.01	61.66
Enumeration	83.82	83.86	65.56	70.29	68.72	74.45
Integer	92.04	78.67	66.07	63.73	58.52	71.80
Number	88.99	89.03	73.61	77.54	17.17	69.27
String	96.19	83.92	84.42	57.57	80.91	80.60
**Evidence Extraction EM - mmBERT**						
**Type**	**BG**	**EL**	**EN**	**PL**	**RO**	**AVG**
Boolean	86.93	87.45	82.20	84.62	83.14	84.87
Date-Time	71.43	73.47	41.76	40.51	14.69	48.37
Enumeration	77.96	72.72	56.24	51.45	59.02	63.48
Integer	89.37	69.41	58.58	53.44	47.67	63.69
Number	77.24	82.91	50.00	61.34	7.53	55.80
String	94.29	77.19	79.66	38.71	72.41	72.45

Table 1: Evidence extraction (S1) performance of mmBERT in the T setting, i.e., using only the original reports in the target language for training, with F1 and EM scores reported separately for each question type.

**Evidence Extraction F1 - mmBERT**						
**Type**	**BG**	**EL**	**EN**	**PL**	**RO**	**AVG**
Boolean	71.95	77.18	48.88	31.08	57.68	57.35
Date-Time	80.91	84.10	57.55	58.45	26.53	61.51
Enumeration	68.38	83.98	52.68	57.61	61.18	64.77
Integer	88.52	78.67	46.34	46.82	51.95	62.46
Number	78.83	89.03	73.61	77.15	18.10	67.34
String	95.83	83.92	87.38	60.16	83.20	82.10
**Evidence Extraction EM - mmBERT**						
**Type**	**BG**	**EL**	**EN**	**PL**	**RO**	**AVG**
Boolean	51.67	57.52	30.84	14.70	36.28	38.20
Date-Time	67.71	68.98	41.76	39.85	11.67	46.00
Enumeration	55.51	72.76	39.01	26.86	47.58	48.35
Integer	84.30	69.41	34.29	31.32	36.92	51.25
Number	50.00	82.91	50.00	60.68	9.68	50.65
String	87.50	77.19	82.46	39.29	74.07	72.10

Table 2: Evidence extraction (S1) performance of mmBERT on possible instances in the T setting, i.e., using only the original reports in the target language for training, with F1 and EM scores reported separately for each question type.

Evidence Extraction F1 - mmBERT						
**Type**	**BG**	**EL**	**EN**	**PL**	**RO**	**AVG**
Boolean	98.17	98.90	98.05	98.46	97.95	98.30
Date-Time	89.66	-	-	45.00	20.00	51.55
Enumeration	96.72	66.67	93.06	93.07	91.97	88.30
Integer	98.91	-	98.44	97.16	82.50	94.25
Number	100.00	-	-	100.00	-	100.00
String	96.30	-	0.00	33.33	50.00	44.91
**Evidence Extraction EM - mmBERT**						
**Type**	**BG**	**EL**	**EN**	**PL**	**RO**	**AVG**
Boolean	98.17	98.90	98.05	98.46	97.95	98.30
Date-Time	89.66	-	-	45.00	20.00	51.55
Enumeration	96.72	66.67	93.06	93.07	91.97	88.30
Integer	98.91	-	98.44	97.16	82.50	94.25
Number	100.00	-	-	100.00	-	100.00
String	96.30	-	0.00	33.33	50.00	53.84

Table 3: Evidence extraction (S1) performance of mmBERT on impossible instances in the T setting, i.e., using only the original reports in the target language for training, with F1 and EM scores reported separately for each question type.

Answer Prediction (S2)

Table 4 reports Accuracy broken down by final answer type for Med42 answer predictions over all test instances. Table 5 reports scores computed only over possible instances, while Table 6 reports scores computed over the remaining instances, i.e., impossible instances.

Type	BG	EL	EN	PL	RO	AVG
Boolean	98.76	99.64	98.91	95.80	99.16	98.45
Date-Time	61.51	82.04	67.65	59.81	72.24	68.65
Enumeration	90.04	94.99	85.47	88.44	92.13	90.22
Integer	79.60	99.77	93.49	93.51	97.81	92.84
Number	60.98	100.00	100.00	81.51	96.77	87.85
String	65.71	42.11	88.14	51.61	55.17	60.55

Table 4: Answer Prediction (S2) performance of Med42 in the T setting, i.e., using only the original reports in the target language for training, with accuracy reported separately for each question type.

Type	BG	EL	EN	PL	RO	AVG
Boolean	98.95	98.70	98.24	97.10	98.89	98.38
Date-Time	69.51	82.04	67.65	67.53	73.75	72.09
Enumeration	97.38	95.07	90.25	92.93	94.07	93.94
Integer	95.89	99.77	90.48	95.98	99.38	96.30
Number	95.31	100.00	100.00	81.20	96.77	94.66
String	50.00	42.11	91.23	57.14	59.26	59.95

Table 5: Answer Prediction (S2) performance of Med42 on possible instances in the T setting, i.e., using only the original reports in the target language for training, with accuracy reported separately for each question type.

Type	BG	EL	EN	PL	RO	AVG
Boolean	98.71	100.00	99.11	95.54	99.25	98.52
Date-Time	0.00	-	-	7.50	0.00	2.5
Enumeration	83.91	83.33	75.28	80.37	87.59	82.10
Integer	47.81	-	98.44	88.64	85.00	79.97
Number	23.73	-	-	100.00	-	61.87
String	70.37	-	0.00	0.00	0.00	17.59

Table 6: Answer Prediction (S2) performance of Med42 on impossible instances in the T setting, i.e., using only the original reports in the target language for training, with accuracy reported separately for each question type.

Evidence-Based Question Answering (S1-S2)

Table 8 reports Accuracy scores broken down by final answer type for Med42 answer prediction using mmBERT-generated evidences instead of gold evidences, reflecting end-to-end performance over all test instances. Table 8 reports scores computed only over possible instances, while Table 9 reports scores computed over the remaining instances, i.e., impossible instances.

**Data Type**	**BG**	**EL**	**EN**	**PL**	**RO**	**AVG**
Boolean	94.69	95.93	92.92	87.35	92.44	92.67
Date-Time	55.16	72.24	34.71	48.87	33.06	48.81
Enumeration	86.98	91.43	74.49	82.33	87.76	84.60
Integer	77.01	95.89	73.96	75.57	92.33	82.95
Number	48.78	94.02	100.00	65.55	67.74	75.22
String	62.86	40.35	86.44	32.26	24.14	49.21

Table 7: Evidence-Based Question Answering (S1-S2) performance in the T setting, i.e., using only the original reports in the target language for training, with accuracy reported separately for each question type. Predicted evidences are generated by mmBERT, while Med42 performs the Answer Prediction (S2).

**Data Type**	**BG**	**EL**	**EN**	**PL**	**RO**	**AVG**
Boolean	85.35	88.24	76.76	49.79	75.30	75.09
Date-Time	62.33	72.24	34.71	53.87	33.75	51.38
Enumeration	91.78	91.59	75.42	84.19	87.99	86.19
Integer	90.47	95.89	60.00	68.97	92.62	81.59
Number	71.88	94.02	100.00	65.81	67.74	79.89
String	37.50	40.35	87.72	32.14	18.52	43.25

Table 8: Evidence-Based Question Answering (S1-S2) performance in the T setting for possible instances, i.e., using only the original reports in the target language for training, with accuracy reported separately for each question type.

**Data Type**	**BG**	**EL**	**EN**	**PL**	**RO**	**AVG**
Boolean	97.33	98.93	97.78	94.88	97.63	97.31
Date-Time	0.00	-	-	15.00	0.00	5.00
Enumeration	82.97	66.67	72.50	78.98	87.23	77.67
Integer	50.73	-	96.88	88.64	90.00	81.56
Number	23.73	-	-	50.00	-	36.87
String	70.37	-	50.00	33.33	100.00	63.43

Table 9: Evidence-Based Question Answering (S1-S2) performance in the T setting for impossible instances, i.e., using only the original reports in the target language for training, with accuracy reported separately for each question type.
