# Supplementary material for: Multilingual Evidence-Based Question-Answering for Stroke Discharge Summaries: Study of Cross-Lingual Heterogeneity in Clinical Reports
Source: J Med Internet Res. 2026 Aug 19;28:e96347. doi: 10.2196/96347 (PMC13489506; doi:10.2196/96347)
Supplement: Supplementary file 4 [file jmir-v28-e96347-s004.pdf]

Error Analysis of Evidence Extraction

Error Type Distribution by Language

Table 1 compares six categories of evidence extraction errors made by the monolingually fine-tuned mmBERT model. The analysis considers all instances where at least one predicted evidence from the list of predicted evidences is not identical to any gold evidence span, or vice versa.

The first category corresponds to cases where the gold evidence list is empty, but the model predicts one or more evidence spans. We denote this category as $Gold = \emptyset$, $Pred \neq \emptyset$. Such errors are generally less severe, as the model provides additional information that can simply be ignored by the second Answer Prediction component. The second category, denoted as $Gold \subset Pred$, includes cases where all gold evidence spans are contained anywhere within the concatenation of predicted evidence spans as substrings, and the gold list is non-empty. In other words, the model successfully identifies all required evidence but additionally extracts some irrelevant information. Conversely, $Pred \subset Gold$ refers to cases where all predicted evidence spans are contained within the gold evidence spans and the prediction is non-empty. Here, the model identifies only part of the required evidence while missing some relevant information. If both the gold and predicted evidence sets are non-empty and share some overlapping content, but neither set is a substring-based subset of the other, the error is categorized as a *Partial Match*. Finally, the category $Gold \neq \emptyset$, $Pred = \emptyset$ represents cases where the model fails to extract any evidence despite the presence of relevant gold evidences.

The results show that approximately 15–36% of all errors belong to the $Gold \subset Pred$ category. In these cases, all required information is present in the prediction, although accompanied by some additional evidence spans. While still counted as errors under F1 and EM evaluation, such cases are relatively less problematic because the relevant information has been successfully identified. An even larger proportion of errors, approximately 23–47%, corresponds to the $Gold \neq \emptyset$, $Pred = \emptyset$ category. These cases indicate that the model failed to identify any relevant evidence despite its presence in the gold annotations, representing the most common error pattern across several languages.

Error type	BG	EL	EN	PL	RO
Pred $\neq \emptyset$, Gold $= \emptyset$	9.4	3.7	6.4	6.8	6.7
Gold $\subset$ Pred	30.0	35.9	15.2	21.3	30.7
Pred $\subset$ Gold	18.1	21.3	17.0	16.0	14.3
Partial match	7.3	10.1	6.5	10.7	9.9
Pred $= \emptyset$, Gold $\neq \emptyset$	30.6	23.1	46.7	39.8	33.5
No match	4.6	5.8	8.2	5.4	4.9

Table 1: Distribution of error types across languages (%).

Error Breakdown by Number of Gold Evidence Spans

Since each question may be associated with multiple evidence spans, Table 2 further analyzes how different types of evidence extraction errors made by the monolingually fine-tuned mmBERT model are distributed with respect to the number of gold evidence spans. Specifically, for instances containing 0, 1, 2, 3, 4, or more than 4 gold evidence spans, we report the average number of predicted evidence spans belonging to each error category.

For each group, we measure how many predicted evidence spans exactly matched a gold evidence span (*Correct*), how many gold evidence spans were substrings of a predicted span ($Gold \subset Pred$), and how many predicted evidence spans were substrings of a gold span ($Pred \subset Gold$). We also report the number of evidence spans classified as *Partial Match*, corresponding to cases where a gold and predicted span share some non-zero token-level overlap but neither span is a substring of the other. Gold evidence spans that do not have any exact or partial match with a predicted span are counted as *Missing*, while predicted evidence spans that do not match any gold evidence span are counted as *Extra*.

Table 3 provides qualitative examples for each error category. The examples use color highlighting to visualize the matching substrings between gold and predicted evidence spans, illustrating how different types of extraction errors arise and how they may affect the final Answer Prediction model.

#Gold	Correct	Extra	Gold⊂Pred	Pred⊂Gold	Partial match	Missing
			**BG**			
0	0.00 ± 0.00	1.24 ± 0.87	0.00 ± 0.00	0.00 ± 0.00	0.00 ± 0.00	0.00 ± 0.00
1	0.13 ± 0.34	0.33 ± 0.63	0.18 ± 0.39	0.12 ± 0.33	0.03 ± 0.18	0.53 ± 0.50
2	0.83 ± 0.70	0.48 ± 0.78	0.11 ± 0.34	0.08 ± 0.27	0.14 ± 0.39	0.84 ± 0.76
3	1.78 ± 0.97	0.72 ± 1.30	0.14 ± 0.43	0.03 ± 0.26	0.07 ± 0.25	0.98 ± 0.97
4	2.21 ± 1.26	0.64 ± 1.17	0.04 ± 0.19	0.11 ± 0.31	0.04 ± 0.19	1.61 ± 1.37
> 4	4.67 ± 2.51	1.36 ± 2.71	0.45 ± 0.89	0.28 ± 0.55	0.24 ± 0.54	1.41 ± 2.20
			**EL**			
0	0.00 ± 0.00	1.54 ± 1.37	0.00 ± 0.00	0.00 ± 0.00	0.00 ± 0.00	0.00 ± 0.00
1	0.10 ± 0.30	0.23 ± 0.46	0.28 ± 0.45	0.19 ± 0.39	0.05 ± 0.21	0.39 ± 0.49
2	0.82 ± 0.74	0.58 ± 0.86	0.27 ± 0.59	0.14 ± 0.43	0.07 ± 0.28	0.70 ± 0.79
3	1.33 ± 1.16	0.49 ± 0.59	0.38 ± 0.80	0.41 ± 0.90	0.21 ± 0.46	0.67 ± 0.83
4	1.85 ± 1.27	1.00 ± 1.56	0.81 ± 1.28	0.19 ± 0.77	0.41 ± 0.83	0.74 ± 1.14
> 4	3.31 ± 3.01	1.18 ± 2.02	0.77 ± 1.80	1.87 ± 3.22	0.36 ± 0.89	0.79 ± 1.96
			**EN**			
0	0.00 ± 0.00	1.22 ± 1.03	0.00 ± 0.00	0.00 ± 0.00	0.00 ± 0.00	0.00 ± 0.00
1	0.05 ± 0.23	0.23 ± 0.60	0.10 ± 0.30	0.14 ± 0.35	0.03 ± 0.16	0.68 ± 0.47
2	0.54 ± 0.65	0.44 ± 1.19	0.10 ± 0.36	0.16 ± 0.41	0.03 ± 0.18	1.17 ± 0.77
3	1.48 ± 0.89	0.94 ± 1.37	0.18 ± 0.46	0.06 ± 0.24	0.12 ± 0.33	1.15 ± 0.96
4	2.29 ± 1.28	1.14 ± 2.50	0.36 ± 0.72	0.14 ± 0.35	0.00 ± 0.00	1.21 ± 1.47
> 4	5.23 ± 3.14	0.81 ± 1.24	0.42 ± 0.69	0.14 ± 0.41	0.00 ± 0.00	1.42 ± 2.42
			**PL**			
0	0.00 ± 0.00	1.44 ± 0.87	0.00 ± 0.00	0.00 ± 0.00	0.00 ± 0.00	0.00 ± 0.00
1	0.11 ± 0.32	0.43 ± 0.84	0.11 ± 0.32	0.14 ± 0.35	0.07 ± 0.25	0.56 ± 0.50
2	0.51 ± 0.69	0.44 ± 0.95	0.16 ± 0.45	0.13 ± 0.42	0.13 ± 0.37	1.07 ± 0.89
3	0.99 ± 1.05	0.66 ± 1.38	0.20 ± 0.54	0.24 ± 0.66	0.34 ± 0.61	1.24 ± 1.30
4	1.48 ± 1.35	0.57 ± 0.93	0.39 ± 0.93	0.07 ± 0.25	0.64 ± 0.87	1.43 ± 1.78
> 4	4.26 ± 6.06	2.02 ± 3.42	0.37 ± 0.81	0.11 ± 0.40	0.82 ± 1.58	3.59 ± 4.90
			**RO**			
0	0.00 ± 0.00	1.20 ± 0.63	0.00 ± 0.00	0.00 ± 0.00	0.00 ± 0.00	0.00 ± 0.00
1	0.05 ± 0.21	0.20 ± 0.47	0.31 ± 0.46	0.11 ± 0.32	0.04 ± 0.20	0.48 ± 0.50
2	0.49 ± 0.62	0.38 ± 0.80	0.26 ± 0.50	0.12 ± 0.34	0.08 ± 0.29	1.05 ± 0.75
3	1.10 ± 1.00	0.41 ± 0.74	0.47 ± 0.81	0.02 ± 0.13	0.08 ± 0.33	1.32 ± 1.13
4	1.74 ± 1.58	0.82 ± 1.77	0.33 ± 0.73	0.21 ± 0.69	0.13 ± 0.40	1.59 ± 1.72
> 4	4.07 ± 2.83	0.48 ± 0.79	0.49 ± 1.09	0.21 ± 0.57	0.13 ± 0.52	2.57 ± 2.55

Table 2: Error decomposition grouped by the number of gold evidence spans. Statistics are reported as mean ± standard deviation over erroneous instances.

Type	Example
**Extra**	**Question:** Was any treatment prescribed at discharge? – Other<br>**Gold:**<br>**Pred:** [adcald3] [alendronic acid] [lactulose] [laxido] [omeprazole] [paracetamol]
	**Question:** Atrial fibrillation/flutter (AF)<br>**Gold:**<br>**Pred:** [af] [atrial fibrillation]
**Gold ⊂ Pred**	**Question:** Is patient's previous medical history known?<br>**Gold:** [mca aneurysm] [htn]<br>**Pred:** [left mca aneurysm] [htn] [gcs 1415]
	**Question:** Is patient's previous medical history known?<br>**Gold:** [hypertension] [asthma] [osteopenia] [bullous pemphigoid] [depression] [alcohol excess]<br>**Pred:** [hypertension] [asthma] [osteopenia] [bullous pemphigoid] [depression] [previous alcohol excess]
**Pred ⊂ Gold**	**Question:** Time of onset of stroke symptoms<br>**Gold:** [noting three days of] [14052018]<br>**Pred:** [three days]
	**Question:** Was any treatment prescribed at discharge?<br>**Gold:** [aspirin] [ramipril] [seretide] [naproxen] [amitriptyline hydrochloride] [atorvastatin] [clopidogrel] [amlodipine] [glyceryl trinitrate] [salbutamol] [tramadol] [amitriptyline]<br>**Pred:** [aspirin] [ramipril] [seretide] [naproxen] [amitriptyline] [atorvastatin] [clopidogrel] [amlodipine] [glyceryl trinitrate] [yl trinitrate] [salbutamol] [tramadol] [amitriptyline]
**Partial match**	**Question:** Old infarcts seen on the imaging?<br>**Gold:** [left parietal and right parietal]<br>**Pred:** [older infarcts in the left parietal] [right parietal lobes] [older asymptomatic infarcts]
	**Question:** Follow-up stroke management appointment scheduled<br>**Gold:** [72 hour tape] [stroke clinic]<br>**Pred:** [outpatient cardiac tape]
**Missing**	**Question:** Is patient's previous medical history known?<br>**Gold:** [alcohol excess] [thrombocytopenia]<br>**Pred:**
	**Question:** Day 1 blood glucose level checks (no. of times)<br>**Gold:** [116] [122]<br>**Pred:**

Table 3: Qualitative error analysis with explicit span-level matching.

Correlation Analysis

Figure 1 presents the Pearson correlation between report length and evidence extraction F1 score, computed across all questions within each report for the predictions produced by the monolingual mmBERT model. The analysis is shown both separately for each language and jointly across all reports from all languages.

For Greek and Romanian, no clear correlation between report length and F1 score can be observed. In contrast, Bulgarian and Polish exhibit a weak negative trend, suggesting that evidence extraction performance slightly decreases as report length increases. English shows the opposite tendency, with longer reports being associated with somewhat higher F1 scores. Overall, however, the observed correlations are small, and the results do not indicate a strong dependence of evidence extraction performance on report length. Across languages, report length appears to have only a limited impact on model performance.

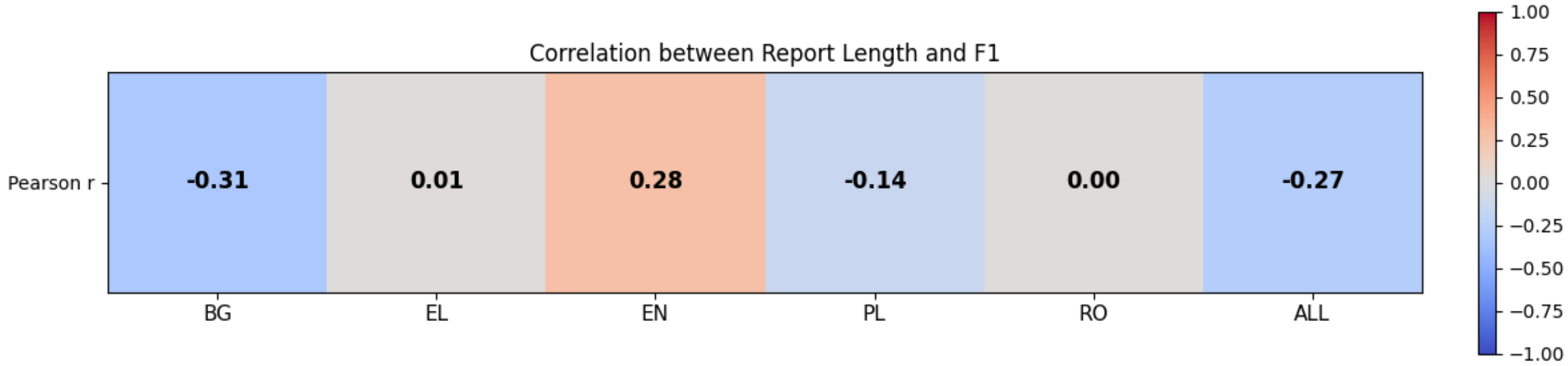

Figure 1: Correlation between report length and F1 score across languages.
