# Supplementary material for: Multilingual Evidence-Based Question-Answering for Stroke Discharge Summaries: Study of Cross-Lingual Heterogeneity in Clinical Reports
Source: J Med Internet Res. 2026 Aug 19;28:e96347. doi: 10.2196/96347 (PMC13489506; doi:10.2196/96347)
Supplement: Supplementary file 5 [file jmir-v28-e96347-s005.pdf]

Answer Prediction Few-Shot Experiments

Table 1 reports results of few-shot experiments comparing various decoder models across all test instances. Table 2 reports scores computed on possible instances only, while Table 3 reports scores computed on the remaining instances, i.e., impossible instances.

	**BG**	**EL**	**EN**	**PL**	**RO**	**AVG**
**0-shot**						
Phi3.5 Mini	43.92	42.43	38.51	37.13	39.17	40.23
MediPhi	**44.92**	**45.70**	**43.34**	40.60	**43.83**	**43.68**
Gemma3	16.72	29.15	25.74	21.39	21.46	22.89
MedGemma	34.67	37.99	34.80	**41.92**	37.23	37.32
Mistral	23.72	32.44	24.44	25.85	31.15	27.52
BioMistral	17.94	37.00	34.54	33.95	23.58	29.40
LLaMA3	25.19	35.07	30.97	23.37	26.34	28.19
Med42	37.17	40.47	39.41	33.95	33.34	36.87
**1-shot**						
Phi3.5 Mini	47.05	**66.74**	57.61	49.60	49.22	54.04
MediPhi	54.32	50.97	45.32	46.69	59.33	51.33
Gemma3	42.56	49.67	43.32	44.78	54.06	46.88
MedGemma	42.66	50.74	42.59	55.11	55.36	49.29
Mistral	23.65	26.43	24.55	26.08	29.90	26.12
BioMistral	38.47	48.84	44.57	46.07	43.59	44.31
LLaMA3	53.78	63.33	53.79	49.77	56.88	55.51
Med42	**62.20**	59.97	**57.63**	**59.72**	**60.26**	**59.96**
**5-shot**						
Phi3.5 Mini	84.43	89.37	84.05	82.97	88.87	85.94
MediPhi	80.60	84.50	78.16	74.96	84.62	80.57
Gemma3	80.00	85.00	76.45	76.72	84.97	80.63
MedGemma	**85.84**	89.67	83.97	**85.98**	**91.60**	**87.41**
Mistral	69.52	77.57	70.60	70.66	80.62	73.80
BioMistral	75.57	83.69	75.41	73.38	79.45	77.50
LLaMA3	80.70	88.75	81.78	77.80	85.61	82.93
Med42	84.83	**91.80**	**85.95**	84.10	90.18	87.37
**20-shot**						
Phi3.5 Mini	89.75	95.14	90.72	87.85	94.13	91.52
MediPhi	89.44	94.69	89.50	87.05	93.90	90.92
Gemma3	89.76	95.63	89.65	87.08	94.40	91.30
MedGemma	**91.47**	**96.44**	91.84	89.28	**95.66**	**92.94**
Mistral	88.06	93.79	89.66	85.52	92.70	89.95
BioMistral	88.96	93.49	87.91	84.99	92.61	89.59
LLaMA3	90.61	96.58	91.20	88.14	94.85	92.27
Med42	90.93	96.03	**92.13**	**89.48**	95.09	92.73

Table 1: Answer Prediction (S2) Accuracy of the generative models under few-shot evaluation with 0, 1, 5, and 20 examples.

	BG	EL	EN	PL	RO	AVG
			**0-shot**			
Phi3.5 Mini	33.07	41.96	41.46	45.02	42.09	40.72
MediPhi	33.11	46.30	48.54	43.78	37.64	41.88
Gemma3	33.57	33.49	31.87	33.81	30.74	32.69
MedGemma	**67.09**	**67.30**	**67.14**	**60.41**	**70.26**	**66.44**
Mistral	49.75	42.45	34.54	43.50	42.54	42.55
BioMistral	39.57	35.55	37.95	39.23	38.56	38.17
LLaMA3	41.76	42.71	39.24	46.37	47.07	43.43
Med42	38.49	42.32	41.81	42.54	43.38	41.71
			**1-shot**			
Phi3.5 Mini	58.32	64.03	57.05	58.65	60.59	59.73
MediPhi	50.87	58.76	53.93	54.98	52.85	54.28
Gemma3	58.94	57.13	47.35	53.23	54.94	54.32
MedGemma	**70.28**	**77.73**	**69.97**	**67.98**	**69.18**	**71.03**
Mistral	54.51	47.38	49.04	53.99	51.24	51.23
BioMistral	54.43	48.23	43.99	49.20	49.52	49.07
LLaMA3	57.99	62.07	52.60	55.18	59.35	57.44
Med42	56.66	69.13	63.58	58.41	54.82	60.52
			**5-shot**			
Phi3.5 Mini	79.10	81.62	74.32	74.24	79.78	77.81
MediPhi	72.19	77.60	68.48	67.38	71.67	71.46
Gemma3	79.14	81.39	67.24	68.82	75.88	74.50
MedGemma	**84.52**	**86.59**	**79.02**	**79.74**	**84.39**	**82.85**
Mistral	80.46	78.84	68.04	71.45	78.49	75.46
BioMistral	77.28	76.59	63.88	64.04	74.12	71.18
LLaMA3	83.40	87.12	75.95	73.92	83.27	80.73
Med42	81.87	86.13	78.72	77.47	83.19	81.48
			**20-shot**			
Phi3.5 Mini	88.53	90.29	84.17	82.62	89.65	87.05
MediPhi	87.79	89.60	82.63	80.34	88.52	85.78
Gemma3	89.94	91.56	82.53	80.66	90.61	87.06
MedGemma	**91.76**	92.71	**87.23**	85.45	**92.58**	**89.95**
Mistral	89.24	89.90	83.52	79.55	88.48	86.14
BioMistral	89.16	87.74	78.97	77.99	87.96	84.36
LLaMA3	91.64	**93.17**	85.55	84.69	91.57	89.32
Med42	90.69	91.79	86.89	**85.61**	91.61	89.32

Table 2: Answer Prediction (S2) Accuracy for possible instances under few-shot evaluation (0, 1, 5, 20 shots).

	BG	EL	EN	PL	RO	AVG
**0-shot**						
Phi3.5 Mini	49.44	42.86	36.78	33.52	37.42	40.00
MediPhi	**50.94**	**45.14**	**40.29**	**39.15**	**47.53**	**44.61**
Gemma3	8.14	25.22	22.15	15.69	15.91	17.42
MedGemma	18.15	11.38	15.82	33.45	17.47	19.25
Mistral	10.46	23.34	18.52	17.76	24.34	18.88
BioMistral	6.92	38.31	32.54	31.53	14.62	24.78
LLaMA3	16.74	28.13	26.12	12.82	13.95	19.55
Med42	36.50	38.79	38.00	30.01	27.34	34.13
**1-shot**						
Phi3.5 Mini	41.30	**69.20**	**57.94**	45.45	42.41	51.26
MediPhi	56.08	43.90	40.26	42.89	63.20	49.27
Gemma3	34.22	42.89	40.96	40.90	53.53	42.50
MedGemma	28.59	26.23	26.53	49.21	47.10	35.53
Mistral	7.93	7.40	10.19	13.30	17.14	11.19
BioMistral	30.34	49.39	44.91	44.63	40.04	41.86
LLaMA3	51.63	64.48	54.48	47.30	55.40	54.66
Med42	**65.02**	51.65	54.14	**60.32**	**63.51**	**58.93**
**5-shot**						
Phi3.5 Mini	**87.14**	96.41	89.75	86.97	94.31	90.92
MediPhi	84.88	90.76	83.83	78.43	92.37	86.05
Gemma3	80.43	88.27	81.86	80.34	90.40	84.26
MedGemma	86.51	92.46	86.88	**88.84**	**95.92**	90.12
Mistral	63.95	76.42	72.10	70.30	81.90	72.93
BioMistral	74.70	90.14	82.18	77.66	82.65	81.46
LLaMA3	79.32	90.23	85.20	79.58	87.01	84.27
Med42	86.34	**96.94**	**90.19**	87.14	94.36	**90.99**
**20-shot**						
Phi3.5 Mini	90.36	99.55	94.57	90.24	96.81	94.31
MediPhi	90.28	99.32	93.53	90.12	97.12	94.07
Gemma3	89.67	99.32	93.82	90.03	96.66	93.90
MedGemma	**91.31**	99.82	94.54	91.03	**97.50**	94.84
Mistral	87.46	97.33	93.27	88.25	95.22	92.30
BioMistral	88.87	98.72	93.15	88.20	95.39	92.87
LLaMA3	90.09	99.67	94.51	89.72	96.81	94.16
Med42	91.06	**99.88**	**95.21**	**91.25**	97.17	**94.91**

Table 3: Answer Prediction (S2) Accuracy for impossible instances under few-shot evaluation (0, 1, 5, 20 shots).
