# Supplementary material for: Multilingual Evidence-Based Question-Answering for Stroke Discharge Summaries: Study of Cross-Lingual Heterogeneity in Clinical Reports
Source: J Med Internet Res. 2026 Aug 19;28:e96347. doi: 10.2196/96347 (PMC13489506; doi:10.2196/96347)
Supplement: Supplementary file 6 [file jmir-v28-e96347-s006.pdf]

## Impact of Extended Context on Answer Prediction

In this appendix, we analyze the robustness of Answer Prediction (S2) Med42 model in the basic $T$ training setting to variations in input context length. The goal is to evaluate whether extending the context beyond compact gold evidence spans (in both training and test sets) affects model performance.

Table 1 reports results over all evaluation instances. Tables 2 and 3 further decompose these results into possible instances, which contain at least one annotated segment with some evidence, and impossible instances, where no evidence is annotated in the input report and therefore no segment is provided.

To test whether this imbalance or structural difference could interact with context length, we construct extended input segments by splitting each report into overlapping windows of $n$ tokens with a stride of 32 tokens. For each instance, the segment containing at least one gold evidence span (if available) is selected as the positive context. For impossible instances, all segments remain negative by construction. Then positive segments are assigned as final segments to the instance.

Despite these differences in supervision structure between possible and impossible cases, we observe that extending the context length from compact evidence spans (approximately 10 tokens) to longer segments (up to 256 tokens) has no meaningful impact on performance. As shown in Table 1, accuracy remains highly stable across all settings, and deviations are negligible. The effect is slightly more pronounced for possible instances, while impossible instances remain unaffected despite being exposed to potentially more heterogeneous and indirectly influential training signals arising from possible cases. Overall, these results indicate that Answer Prediction (S2) is insensitive to moderate increases in input context length.

| **Context Type** | **BG** | **EL** | **EN** | **PL** | **RO** | **AVG** |
|---|---|---|---|---|---|---|
| Evidences (∼10 tok.) | **93.04** | **97.82** | **94.88** | **92.75** | **96.91** | **95.08** |
| 64 tok. segments | 92.83 | 97.48 | 93.85 | 92.46 | 96.59 | 94.64 |
| 128 tok. segments | 92.64 | 97.56 | 93.38 | 92.19 | 96.55 | 94.46 |
| 192 tok. segments | 92.60 | 97.46 | 93.36 | 92.26 | 96.14 | 94.36 |
| 256 tok. segments | 92.83 | 97.39 | 93.51 | 91.99 | 96.11 | 94.37 |

Table 1: Answer Prediction (S2) accuracy of the Med42 model using gold evidences and report segments of increasing length (64–256 tokens), where each segment contains the corresponding evidences.

| **Context Type** | **BG** | **EL** | **EN** | **PL** | **RO** | **AVG** |
|---|---|---|---|---|---|---|
| Evidences (∼10 tok.) | **94.95** | **95.45** | **92.03** | **91.27** | **94.78** | **93.70** |
| 64 tok. segments | 94.33 | 94.74 | 89.06 | 90.27 | 93.66 | 92.41 |
| 128 tok. segments | 93.54 | 94.93 | 87.93 | 89.59 | 93.38 | 91.87 |
| 192 tok. segments | 93.63 | 94.77 | 87.78 | 89.27 | 92.42 | 91.57 |
| 256 tok. segments | 93.87 | 94.54 | 88.27 | 88.56 | 92.46 | 91.54 |

Table 2: Answer Prediction (S2) accuracy on possible instances using the Med42 model with gold evidence spans and report segments of increasing length (64–256 tokens), each containing the corresponding evidence.

| **Context Type** | **BG** | **EL** | **EN** | **PL** | **RO** | **AVG** |
|---|---|---|---|---|---|---|
| Evidences (∼10 tok.) | 92.07 | 99.97 | 96.55 | 93.42 | 98.18 | 96.04 |
| 64 tok. segments | 92.07 | 99.97 | **96.66** | 93.46 | 98.34 | 96.10 |
| 128 tok. segments | 92.18 | 99.94 | 96.57 | 93.39 | **98.44** | 96.10 |
| 192 tok. segments | 92.07 | 99.91 | 96.63 | **93.62** | 98.37 | 96.12 |
| 256 tok. segments | **92.30** | **99.97** | 96.57 | 93.57 | 98.30 | **96.14** |

Table 3: Answer Prediction (S2) accuracy of the Med42 model using gold evidences and report segments of increasing length (64–256 tokens), where each segment contains the corresponding evidences for impossible instances.
